# HERZCHECK: Early Detection of Subclinical Heart Failure Using Mobile Cardiac Magnetic Resonance and Telemedicine in Rural and Underressourced Regions

**DOI:** 10.1161/CIRCHEARTFAILURE.125.013935

**Published:** 2026-08-04

**Authors:** Sebastian Kelle, Maximilian Leo Müller, Rebecca Elisabeth Beyer, Gisela Thiede, Anna Clara Nolden, Henning Steen, Bjoern Andrew Remppis, Johannes Wieditz, Alex Tuit, Mina Cvetkovic, Patrick Doeblin, Undine Ella Witt, Florian André, Sein Schmidt, Alexander Huppertz, Dusan Simic, Dirk Müller, Arim Shukri, Katrin Christiane Reber, Ulf Landmesser, Norbert Frey, Burkert Pieske, Eike Nagel, Stephanie Stock, Volkmar Falk, Hannah Kentenich, Tim Friede

**Affiliations:** 1 Department of Cardiology, Angiology and Intensive Care Medicine (S.K., M.L.M., R.E.B., G.T., A.C.N., A.T., M.C., P.D., U.E.W., B.P., U.L.), Deutsches Herzzentrum der Charité, Berlin, Germany.; 2 Department of Cardiology, Angiology, and Intensive Care (U.L.), Deutsches Herzzentrum der Charité, Berlin, Germany.; 3 Charité - Universitätsmedizin Berlin, Corporate Member of Freie Universität Berlin and Humboldt-Universität zu Berlin, Germany (S.K., M.L.M., R.E.B., G.T., A.C.N., A.T., M.C., P.D., U.E.W., S. Schmidt, V.F.).; 4 DZHK (German Centre for Cardiovascular Research), Berlin, Germany (S.K., M.L.M., R.E.B., A.C.N., P.D., V.F.).; 5 Department of Cardiology, Angiology and Pneumology, University Hospital Heidelberg/Mannheim, Germany (H.S., F.A., N.F.).; 6 DZHK (German Centre for Cardiovascular Research), Heidelberg, Germany (H.S., N.F.).; 7 Heart and Vascular Center Bad Bevensen, Germany (B.A.R.).; 8 Department of Medical Statistics, University Medical Center Göttingen, Germany (J.W., T.F.).; 9 Institute for Health Economics and Clinical Epidemiology (IGKE), Faculty of Medicine and University Hospital Cologne, University of Cologne, Germany (D.S., D.M., A.S., S. Stock, H.K.).; 10 Herzinstitut Berlin, Kardiologische Gemeinschaftspraxis, Germany (U.E.W.).; 11 AOK Nordost. Die Gesundheitskasse, Health Services Management, Berlin, Germany (K.C.R.).; 12 Department of Cardiothoracic and Vascular Surgery, Deutsches Herzzentrum der Charité (DHZC), Berlin, Germany (V.F.).; 13 University of Potsdam, University Outpatient Clinic, Sports Medicine and Sports Orthopaedics, Germany (A.H.).; 14 Berlin Institute of Health (BIH) at Charité – Universitätsmedizin Berlin, Germany (S. Schmidt).; 15 Department of Neurology, Charité-Universitätsmedizin Berlin, corporate member of the Freie Universität Berlin and Humboldt-Universität Berlin, Germany (S. Schmidt).; 16 Center for Stroke Research (CSB), Charité-Universitätsmedizin Berlin, Corporate Member of the Freie Universität Berlin and Humboldt-Universität Berlin, Germany (S. Schmidt).; 17 Division of Cardiology, University Medicine Rostock, Germany (B.P.).; 18 Institute for Experimental and Translational Cardiovascular Imaging, DZHK Centre for Cardiovascular Imaging, Goethe University Frankfurt, Germany (E.N.).; 19 DZHK (German Centre for Cardiovascular Research), Göttingen, Germany (T.F.).

**Keywords:** cardiovascular diseases, heart failure, magnetic resonance imaging, mobile health units, risk factors, sprains and strains, telemedicine

## Abstract

**BACKGROUND:**

There is a growing consensus that screening for subclinical preheart failure (HF) may reduce the burden of symptomatic HF by allowing for timely preventive interventions. However, validated screening algorithms are currently lacking. The aim of this study was to evaluate a fully mobile telemedically supervised cardiac magnetic resonance, as a simple standardized, and ubiquitously applicable screening algorithm for subclinical pre-HF in rural and underresourced regions.

**METHODS:**

HERZCHECK was a cross-sectional cohort study conducted at 12 sites across rural and underresourced regions of Germany. Asymptomatic participants (40–69 years) with ≥1 cardiovascular risk factor—obesity, smoking, arterial hypertension, diabetes, hypercholesterolemia, or chronic kidney disease—were enrolled and underwent telemedically supervised contrast-free short cardiac magnetic resonance in mobile screening units. Subclinical pre-HF was diagnosed using a predefined cutoff of global longitudinal strain ≥−15%. A matched and entropy-balanced control cohort constructed from claims data was used to determine the time difference between detection of subclinical pre-HF in HERZCHECK and the first symptom-based diagnosis of HF within the standard of care.

**RESULTS:**

Between June 2021 and April 2023, 4666 participants were enrolled in the study, of which 4509 participants were included in the final analysis. The prevalence of subclinical pre-HF in the studied at-risk population was 22.7% (95% CI, 21.5%–23.9%). Global longitudinal strain-based screening identified subclinical pre-HF 6.7 years before the average onset of symptomatic HF within the standard of care (n=8420).

**CONCLUSIONS:**

Subclinical pre-HF affects approximately one-fourth of the at-risk population in rural and underresourced regions. The HERZCHECK approach identifies patients suitable for targeted preventive interventions ≈7 years earlier than the standard of care.

**REGISTRATION:**

URL: https://www.clinicaltrials.gov; Unique identifier: NCT05122793.

**WHAT IS NEW?**

- HERZCHECK demonstrates the feasibility of a standardized, fully mobile and telemedicine-supervised cardiac magnetic resonance-based screening approach using global longitudinal strain as a scalable end point to detect subclinical preheart failure (HF).
- Systematic application of this strategy identifies subclinical pre-HF in 22.7% of the rural at-risk population and ≈7 years earlier than the diagnosis of symptomatic HF under the current standard of care.

**WHAT ARE THE CLINICAL IMPLICATIONS?**

- On-demand mobile and telemedicine-supervised cardiac magnetic resonance might support the clinical needs of patients in rural and underresourced areas to improve healthcare and support local physicians.
- Integration of innovative systematic screening into routine care could substantially advance the timely identification of subclinical pre-HF and enable earlier initiation of strategies to prevent progression to symptomatic disease.
- Given the current lack of consensus, further studies are required to define the optimal management of subclinical pre-HF and to determine whether the diagnostic lead time achieved ultimately translates into improved clinical outcomes.

Strategies to reduce the socioeconomic burden of heart failure (HF) are much sought after, especially in rural and underresourced regions affected by a strong imbalance between high prevalence and insufficient access to specialized healthcare resources.^1,2^ Results from the STOP-HF and PONTIAC trials suggest that the early identification of subclinical pre-HF among at-risk patients reduces the incidence of clinically manifest HF by allowing for timely, targeted preventive interventions.^3,4^ However, suitable standardized screening approaches are currently unavailable in clinical routine care.

Signs of systolic dysfunction may be the optimal screening target, as indicated by a meta-analysis involving >25 000 participants, which revealed that asymptomatic left ventricular systolic dysfunction is associated with an almost 5-fold relative risk of transitioning to clinically manifest HF in maximally adjusted models.^5^ Although being the classic domain of the left ventricular ejection fraction (LVEF), myocardial deformation imaging has emerged as a more robust, reproducible, and sensitive method to detect subtle alterations in systolic function. Among the different myocardial deformation indices, recent evidence suggests that left ventricular global longitudinal strain (GLS), in particular, is a precise predictor of incident HF among the general or at-risk population.^6–8^ Consequently, GLS appears to be a promising marker to identify subclinical pre-HF and guide targeted lifestyle or pharmacological interventions to prevent the transition to clinically manifest HF.

Leveraging these favorable characteristics of GLS, HERZCHECK sought to accurately determine the prevalence of subclinical pre-HF in an at-risk population and to assess whether GLS can improve the early detection of patients who develop clinically manifest HF when utilized as part of a standardized screening routine. Through the use of mobile, telemedically supervised cardiac magnetic resonance (CMR) units, the evaluated screening routine was specifically developed as an innovative solution to address the needs of rural and underresourced regions while ensuring maximum diagnostic accuracy and reproducibility.

## Methods

### Data Availability

The data supporting the findings of this study are not openly available but may be shared upon reasonable request to the corresponding author and with the approval of the HERZCHECK consortium. Data will only be shared with academic institutions after signing a data-sharing agreement.

### Study Design and Participants

HERZCHECK was an investigator-initiated, cross-sectional cohort study with an integrated prospective randomized controlled trial that was conducted at 12 sites across rural and underresourced regions in Germany under telemedical supervision of the Deutsches Herzzentrum der Charité between October 2020 and September 2024. Regions were classified as rural and underresourced based on the official definition used by the German Federal Ministry of Education and Research.^9^ The study was approved by the ethics committee of the Charité-Universitätsmedizin Berlin, Germany (EA4/204/20), registered on ClinicalTrials.gov (REGISTRATION: URL: https://www.clinicaltrials.gov; Unique identifier: NCT05122793), and conducted in accordance with the Declaration of Helsinki and relevant guidelines for good clinical practice. All participants provided written informed consent before inclusion in the study. A comprehensive summary of the study protocol has previously been published.^10^

The herein presented cross-sectional cohort study had 2 primary objectives: first, to accurately determine the prevalence of subclinical pre-HF by the criterion of a reduced GLS in a representative at-risk population residing in rural and underresourced regions in Germany; and second, to test the hypothesis that a standardized systematic GLS-based screening enables the identification of patients who develop HF significantly earlier than symptomatic HF would have been diagnosed within the current standard of care. Additional exploratory objectives were to assess the association between specific risk factor constellations and the prevalence of subclinical pre-HF, as well as the association of subclinical pre-HF with adverse events.

To determine the prevalence of subclinical pre-HF, consecutive individuals presenting to 1 of the 12 study sites were assessed for eligibility for participation by a study physician via telemedical consultation. Individuals between 40 and 69 years of age and with existing statutory health insurance were eligible to participate in HERZCHECK if they had at least one of the following modifiable cardiovascular risk factors: hypercholesterolemia, arterial hypertension, obesity, smoking/tobacco consumption, chronic diabetes, or chronic kidney disease. Individuals were excluded if they had previously diagnosed HF or symptoms related to HF. Upon inclusion in the study, all participants underwent contrast- and stress-agent-free CMR. Subclinical pre-HF was diagnosed using a previously defined cutoff of the GLS (ie, GLS ≥−15%).^11^ Additional baseline examinations included a standardized medical history, validated quality-of-life questionnaires, and laboratory testing.

The potential of the proposed GLS-based screening to enable the identification of patients with subclinical pre-HF earlier than the onset of symptomatic HF within the standard of care was assessed by comparing a subgroup of the HERZCHECK cohort, including participants diagnosed with subclinical pre-HF, with a control cohort constructed from claims data of a major health insurance company in Germany (AOK Nordost – Die Gesundheitskasse, Potsdam, Germany). To be eligible for inclusion in the HERZCHECK subgroup, participants had to be insured with the same aforementioned health insurance company, reside in Mecklenburg-Pomerania or Brandenburg at the end of the year in which the CMR was performed, and have consented to the analysis of their claims data. Initial matching (1:10) and subsequent entropy balancing (details provided as Supplemental Methods S1 and Table S1) were conducted to ensure comparability of the control cohort with the HERZCHECK subgroup regarding cardiovascular risk profile. Additional approval for the use of the claims data was obtained from the ethics committee of the University of Cologne, Germany (EA23-1238).

### Procedures

The following provides comprehensive descriptions of the study procedures that are of central relevance for the interpretation of the data reported in this article. Details regarding all other study procedures, including a telemedical consultation to collect participant-reported data on sex, medical history, and current medication, on-site measurement of vital parameters, a blood draw for laboratory testing, and self-administered quality-of-life questionnaires, have previously been published.^10^

#### Cardiac Magnetic Resonance

All participants in the HERZCHECK cohort underwent a stress- and contrast-agent-free CMR exam that was performed by an extensively trained CMR technician in a fully mobile CMR unit equipped with a 1.5T Philips Ingenia dStream scanner (Philips Healthcare, Best, the Netherlands). The standardized protocol was designed according to current recommendations of the Society of Cardiac Magnetic Resonance and included a survey in 3 dimensions, short-axis, 2-, 3-, and 4-chamber cine sequences, as well as T1- and T2-mapping sequences. Images were sent to the Deutsches Herzzentrum der Charité, where they were analyzed offline by experienced Society of Cardiac Magnetic Resonance-certified study investigators using cvi42, version 5.13.7 (Circle Cardiovascular Imaging, Inc, Calgary, Canada). Left ventricular volumes and function were quantified from the full short-axis stack following current Society of Cardiac Magnetic Resonance consensus recommendations for the standardized image interpretation and postprocessing in CMR.^12^ T1- and T2-relaxation times were measured in the midventricular septum. Semiautomatic feature tracking was used to analyze the GLS from 3 long-axis views, with manual selection of identical end-diastolic and end-systolic phases across short-axis and long-axis slices. When automated contours did not correctly align with the endocardial and epicardial borders, delineation was adjusted to ensure exclusion of trabeculae and papillary muscles. Incomplete CMR data sets precluding valid analysis of the GLS led to exclusion of the respective participant from further analyses.^10^

#### Identification of Adverse Events in the HERZCHECK Cohort

In line with the integrated randomized controlled trial, all participants from the HERZCHECK cohort who were diagnosed with subclinical pre-HF and 10% of those with preserved GLS were followed for 12 months using structured questionnaires.^10^ In addition, for a subgroup of participants insured with the cooperating health insurance provider (AOK Nordost–Die Gesundheitskasse, Potsdam, Germany), claims data for each participant’s specific period of 24 months before and 12 months after baseline (minimum 9 months for outpatient visits) were available. Based on this, adverse events within 12 months after baseline could be investigated. Adverse events considered for this analysis included all-cause death, HF-related hospitalizations, and HF-related events (composite of HF-related hospitalizations and outpatient events). The respective definitions are provided as Supplemental Methods S2.

#### Quantification of Time Gained for Prevention of Symptomatic HF

To quantify the time gained for prevention of symptomatic HF, the difference between the age at subclinical pre-HF detection (ie, date of CMR with impaired GLS) in participants of the HERZCHECK cohort and the age at first symptomatic HF diagnosis in the matched (1:10) and entropy-balanced control cohort was analyzed (details provided as Supplemental Methods S3). The age at HF detection in the control cohort was defined as the age at first validated diagnosis of incident HF, as documented in claims data spanning a 9-year observation period (2014–2022). The respective definitions of the diagnoses qualifying as validated incident HF are summarized in Supplemental Methods S4. The frequency and incidence rate per 1000 patient years of symptomatic HF in the control cohort were also calculated.

### Statistical Analysis

Statistical analyses were conducted on data from all eligible participants extracted after database lock using R, version 4.3.3 or higher (R Foundation for Statistical Computing, Vienna, Austria). Continuous and categorical data are presented as mean±SD and counts with corresponding percentages, respectively. Effect measures are given with corresponding 95% CIs. All hypothesis tests were performed 2-sided at a significance level of 5%.

To determine the prevalence of subclinical pre-HF, the proportion of participants with GLS ≥−15% is reported along with Clopper-Pearson 95% CIs. With the sample size of 4509 participants, an accuracy (half-width of the 95% CI) of ±1% at an assumed prevalence of 20% of subclinical pre-HF can be achieved. Dedicated sample size calculations have previously been published.^10^

Demographic data, as well as data pertaining to secondary or exploratory end points, are summarized for the entire cohort and compared between participants with subclinical pre-HF (ie, GLS ≥−15%) and those with preserved GLS using *t* tests or χ^2^ tests, as appropriate. Nonnormally distributed parameters reflecting concentrations (ie, NT-proBNP [N-terminal pro-B-type natriuretic peptide] and ferritin) were log-transformed beforehand. Secondary, exploratory, and safety outcomes were not adjusted for multiple testing due to their exploratory nature. Missing values were excluded from the analysis on a per-parameter basis and were not subject to imputation.

The association of individual self-reported cardiovascular risk factors (ie, inclusion criteria) with subclinical pre-HF by the criterion of GLS ≥−15% was assessed using a multivariable logistic model including age decades (40–49 years, 50–59 years, 60–69 years), sex (female, male), obesity (yes, no), arterial hypertension (yes, no), diabetes (yes, no), hypercholesterolemia (yes, no), smoking (yes, no), and chronic kidney disease (yes, no) as covariates.

Adverse event-free survival was estimated for a subgroup of HERZCHECK participants with available claims data using Kaplan-Meier analyses. Differences in adverse event-free survival between participants with subclinical pre-HF and those with preserved GLS were assessed using log-rank tests (details provided as Supplemental Methods S2).

Analyses to quantify the time gained for prevention were performed on a subgroup of all HERZCHECK participants who were diagnosed with subclinical pre-HF and were insured with the cooperating health insurance provider (AOK Nordost–Die Gesundheitskasse, Potsdam, Germany), as well as the 1:10 matched and entropy-balanced control cohort. Baseline characteristics (obtained from claims data) are reported and were compared between the subgroup of HERZCHECK participants and the control cohort using absolute standardized mean differences. The frequency and incidence rate per 1000 patient-years of symptomatic HF in the control cohort were calculated based on available claims data. The time gained for prevention is reported as the mean difference with 95% CI between the age at detection of subclinical pre-HF in HERZCHECK and the age at first incident of symptomatic HF in the control cohort, with statistical significance being tested using a weighted *t* test (details provided as Supplemental Methods S3). Several sensitivity analyses and prespecified subgroup analyses according to sex, age, region of residence, and severity of GLS impairment were performed to assess the robustness of the obtained results (details provided as Supplemental Methods S5). The Benjamini-Hochberg procedure was applied to account for multiple testing.

### Role of the Funding Source

The HERZCHECK trial was fully funded by the Innovation Fund for the promotion of new forms of care of the Federal Joint Committee (Gemeinsamer Bundesausschuss; Funding Reference: 01NVF19014). The funder of the study had no role in the study design, data collection, data analysis, data interpretation, or writing of the report.

## Results

Between June 2021 and April 2023, 4784 individuals presenting to 1 of the 12 study sites were screened. After exclusion of 118 individuals who did not meet the eligibility criteria, 4666 participants were enrolled. Another 151 enrolled participants were excluded due to incomplete CMR examinations (n=139, claustrophobia; n=12, insufficient image quality), and 6 participants were excluded due to technical issues, resulting in a total of 4509 participants included in the final analysis (flowchart provided as Figure S1).

Clinical characteristics of the HERZCHECK population are summarized in Table 1. Of all participants, 2511 (56%) were female and 1998 (44%) were male. The mean age was 60±7 years. In descending order, the most common participant-reported cardiovascular risk factors at inclusion were arterial hypertension (74%; n=3349), smoking (59%; n=2633), hypercholesterolemia (47%; n=2101), obesity (44%; n=1989), diabetes (21%; n=957), and chronic kidney disease (5%; n=208). Notably, 2083 (46%) participants reported having ≥3 of the cardiovascular risk factors prespecified for inclusion. Only 296 (7%) and 154 (4%) participants reported a history of coronary heart disease or myocardial infarction, respectively.

**Table 1.**
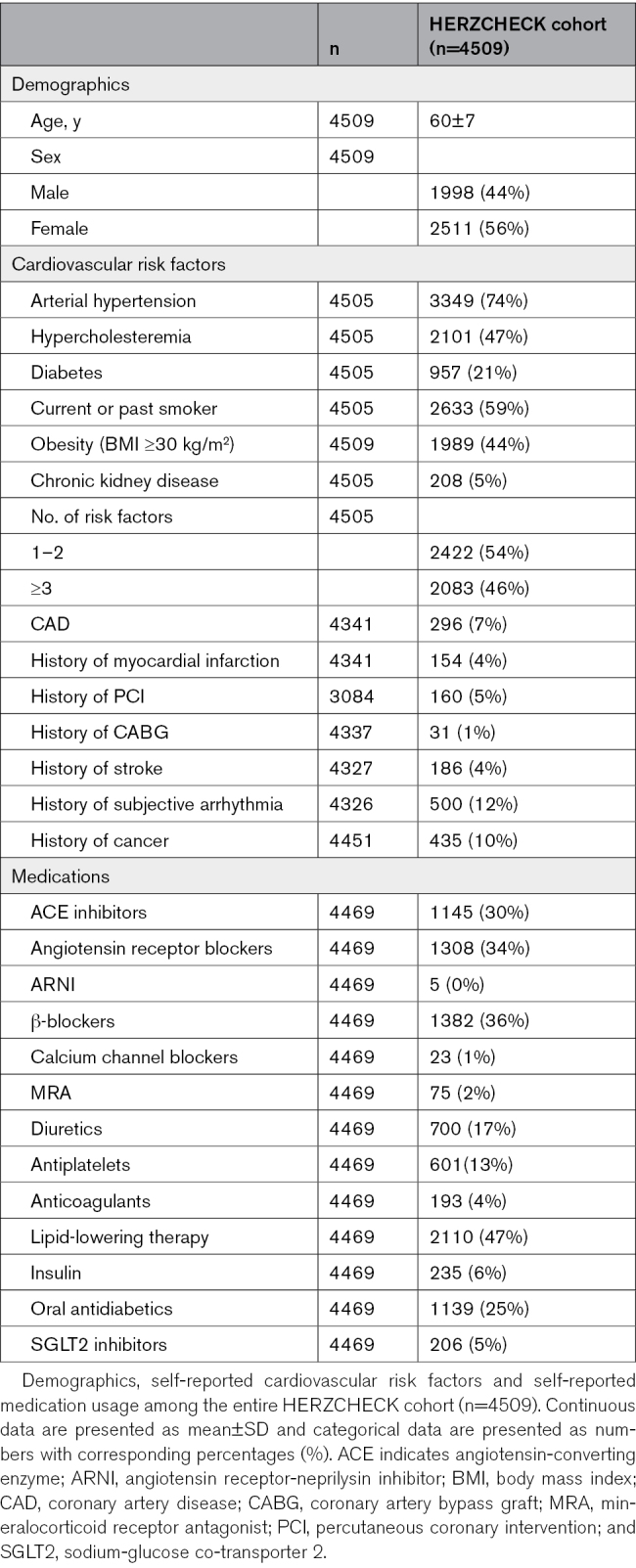
Demographics of the HERZCHECK Cohort

**Figure 1.**
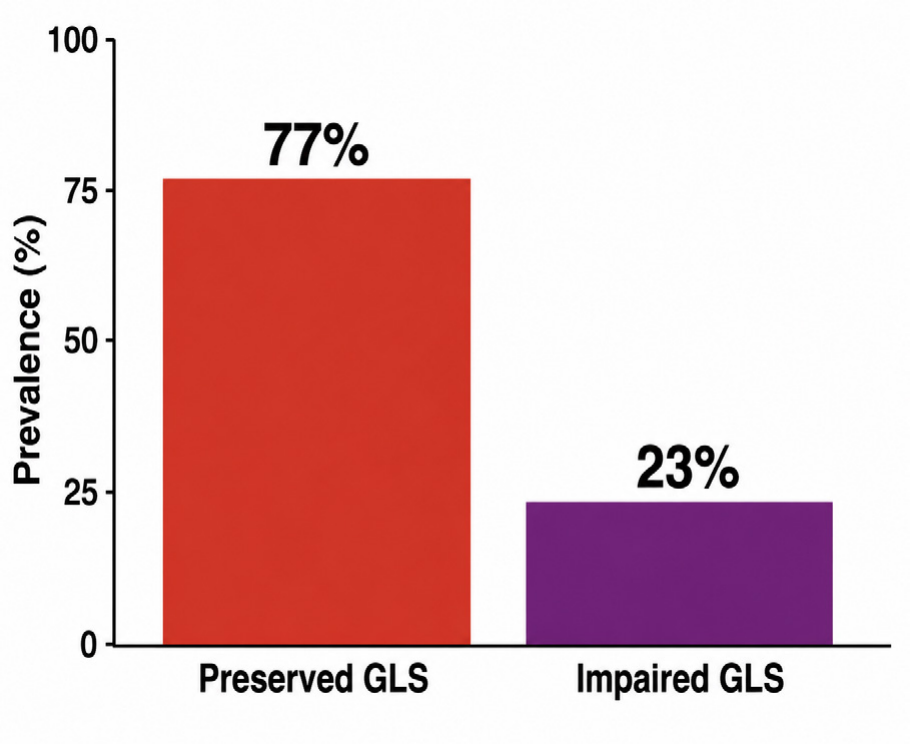
**Overall prevalence of impaired global longitudinal strain (GLS).** Prevalence of preserved (77.3%; red bar) and impaired (22.7%; purple bar) GLS in the HERZCHECK cohort (n=4509).

With a GLS ≥−15% being identified in a total of 1023 participants, the prevalence of subclinical pre-HF in the studied at-risk population was 22.7% (95% CI, 21.5%–23.9%; Figure 1). The prevalence of subclinical pre-HF was significantly higher in male participants compared with female participants (37% versus 12%; *P*<0.001) and slightly elevated in the highest age decade compared with younger participants (40–49 years, 21%; 50–59 years, 20%; 60–69 years, 25%; *P*=0.002). Regional variation in the prevalence of subclinical pre-HF is shown in Figure S2, with the lowest and highest prevalence being 19% and 27%, respectively. Of those diagnosed with subclinical pre-HF by the criterion of an impaired GLS, 278 (32%) individuals had elevated NT-proBNP plasma levels (ie, ≥14.78 pmol/L), and 244 (24%) had a reduced LVEF (ie, <50%). Additional CMR findings, as well as data from laboratory tests and quality-of-life questionnaires, are summarized and compared between participants with and without subclinical pre-HF in Table 2. Cross-tables demonstrating the relationship between GLS-based subclinical pre-HF and elevated NT-proBNP or reduced LVEF are provided as Tables S2 and S3. Notably, GLS-based subclinical pre-HF would have been missed in 15.6% (n=586/3762) and 17.3% (n=779/4509) of all participants based on NT-proBNP or LVEF, respectively. A proportional Venn-diagram visualizing the relationship between impaired GLS-based subclinical pre-HF, elevated NT-proBNP and reduced LVEF is provided as Figure S4.

**Table 2.**
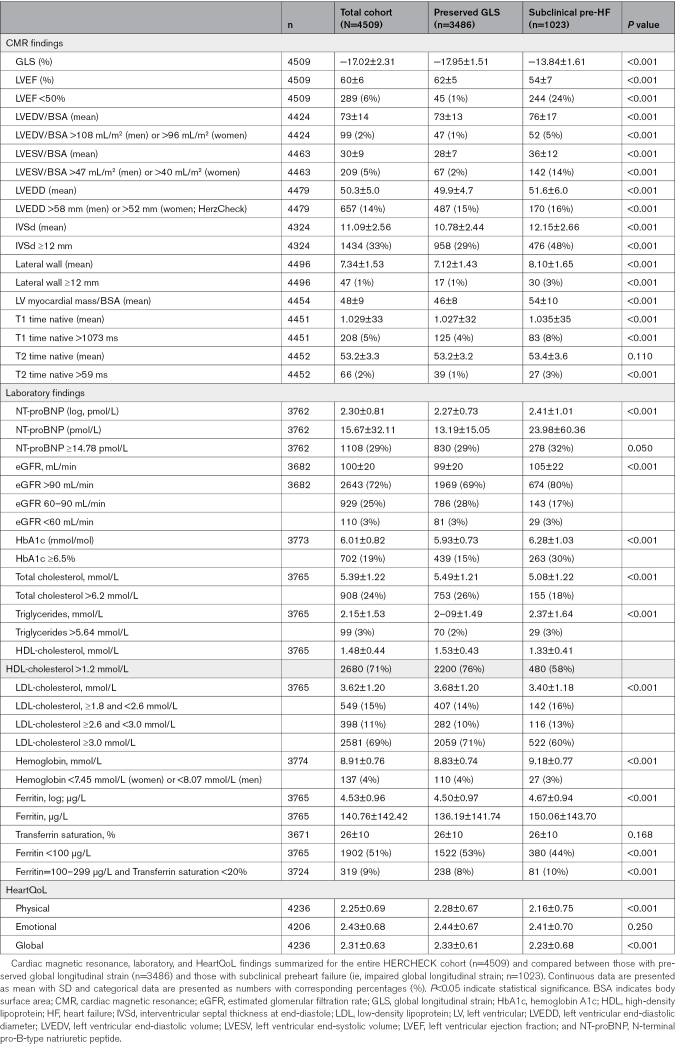
CMR, Laboratory, and HeartQol Findings of the HERZCHECK Cohort

**Figure 2.**
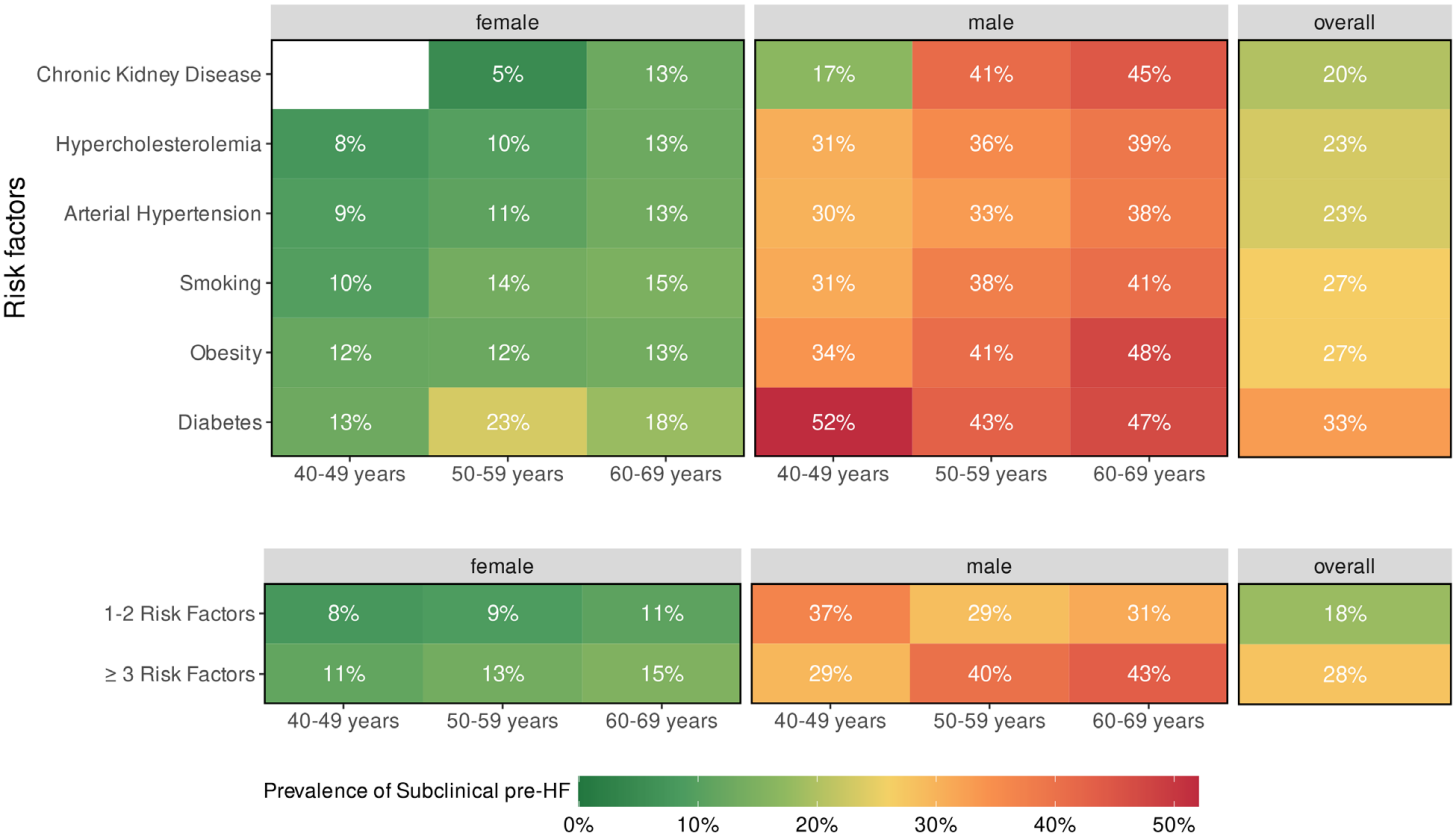
**Prevalence of subclinical heart failure (HF) and risk factor constellations.** Observed prevalence of subclinical pre-HF (ie, impaired global longitudinal strain) in different risk factor constellations.

Beyond male sex (odds ratio, 4.18 [95% CI, 3.58–4.89]; *P*<0.001), cardiovascular risk factors independently associated with the presence of subclinical pre-HF in the multivariable logistic regression analysis included smoking (odds ratio, 1.39 [95% CI, 1.19–1.63]; *P*<0.001), obesity (odds ratio, 1.49 [95% CI, 1.28–1.73]; *P*<0.001), and diabetes (odds ratio, 1.71 [95% CI, 1.44–2.04]; *P*<0.001). No significant independent associations with subclinical pre-HF were identified for increasing age decades, arterial hypertension, hypercholesterolemia, or chronic kidney disease (Figure S4). The observed prevalence of subclinical pre-HF in HERZCHECK participants with different cardiovascular risk factor constellations is reported in Figure 2.

**Figure 3.**
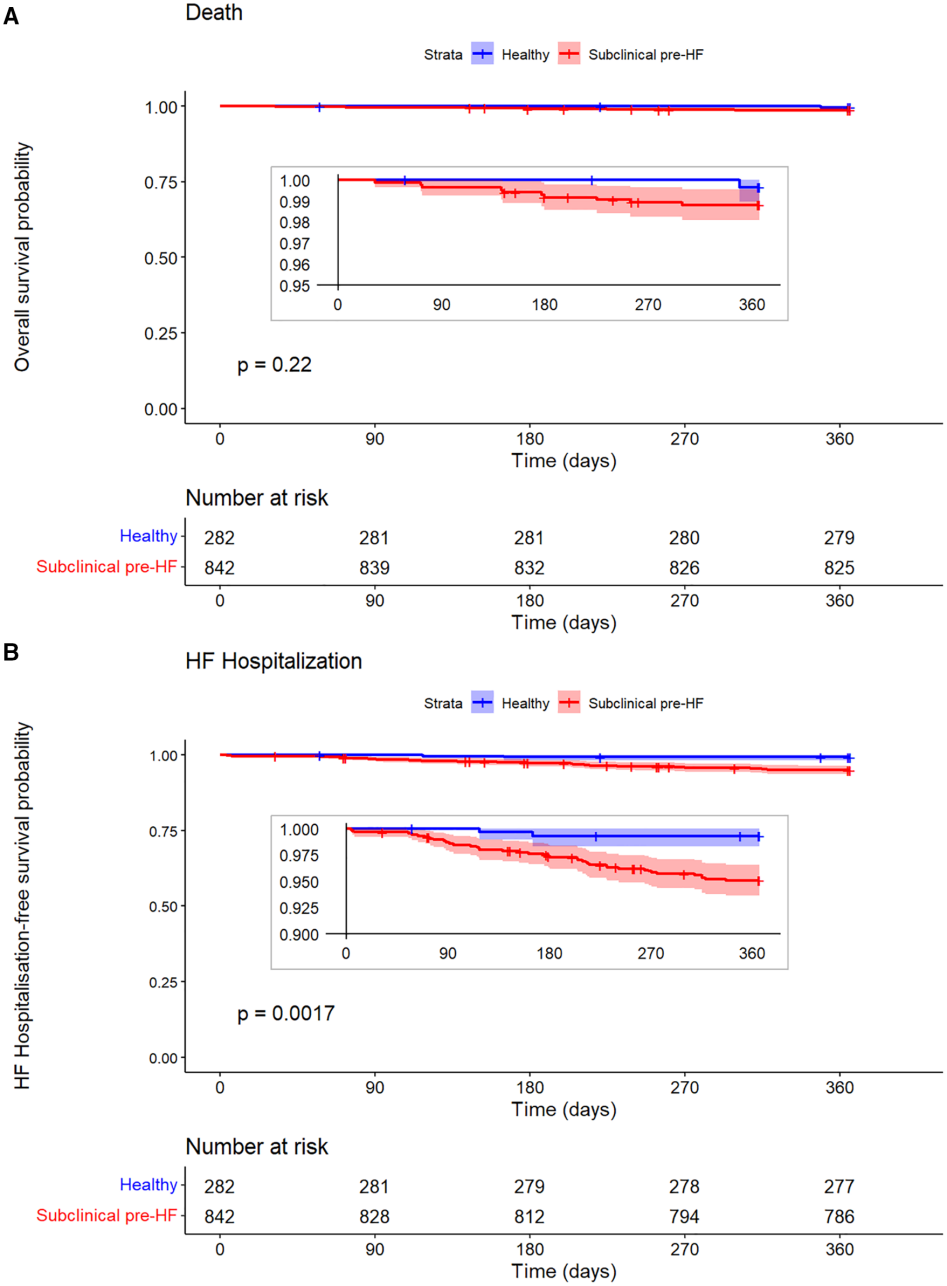
**Kaplan-Meier-curves of HERZCHECK participants.** Kaplan-Meier curves comparing time to (**A**) all-cause death and (**B**) first heart failure (HF) hospitalization between healthy participants (ie, preserved global longitudinal strain; blue lines) and those diagnosed with subclinical pre-HF (ie, impaired global longitudinal strain; red lines).

Claims data for the 1-year follow-up period were available for 1124 HERZCHECK participants (n=878 with 12 months of data on outpatient events), including 282 with preserved GLS (n=221 with 12 months of data on outpatient events) and 842 with subclinical pre-HF (n=657 with 12 months of data on outpatient events). The 1-year incidence of all-cause death, HF-related hospitalizations, and HF-related events was 1% (n=11), 4% (n=43), and 15% (n=135), respectively (details provided as Table S4). No differences in overall survival were observed between those with preserved GLS and those with subclinical pre-HF (*P*=0.22). However, participants with subclinical pre-HF had a significantly increased risk of HF-related hospitalizations (*P*=0.0017). Kaplan-Meier curves illustrating these comparisons are presented in Figure 3 and Figure S5.

Of all participants in the HERZCHECK cohort diagnosed with subclinical pre-HF, 842 (82%) were eligible for inclusion in the subgroup that was compared against the control cohort (n=8420, ratio 1:10; flowchart provided as Figure S1). After entropy balancing, there were no differences in baseline characteristics between the HERZCHECK subgroup and the control cohort (absolute standardized mean difference <0.1 for all characteristics; comparisons of the baseline characteristics of both groups before and after entropy balancing are presented in Tables S5 and S6; Figure S6). Based on the available claims data spanning a 9-year observation period, a weighted 26% (n=2177) and 20% (n=1665) of the control cohort were diagnosed with symptomatic HF or died, respectively. At an overall weighted observation time of 60 457.9 patient-years (mean, 7.2±2.8 years/patient), this translated to a weighted HF incidence of 36.0 per 1000 patient-years.

**Figure 4.**
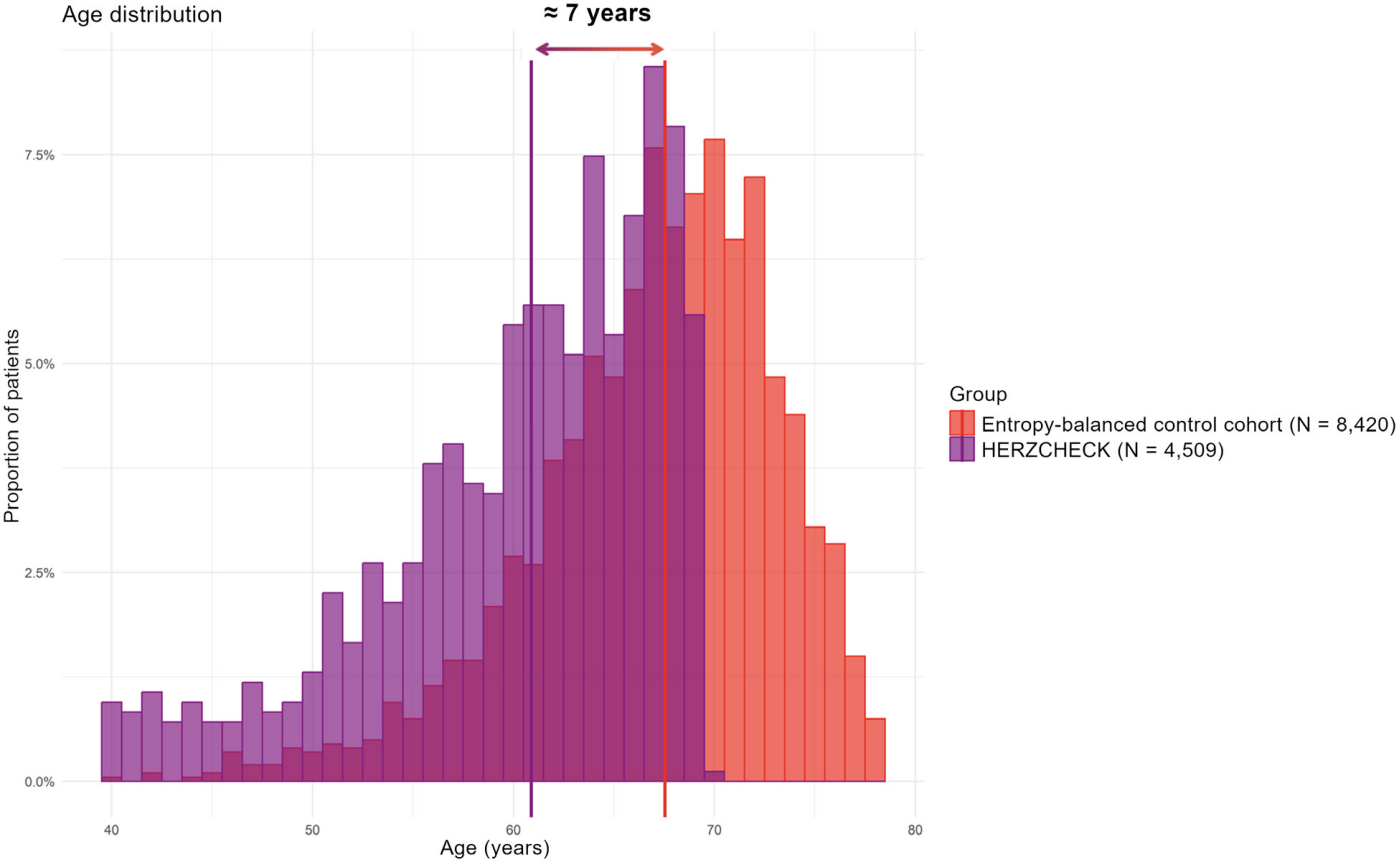
**Time gained for prevention in participants with subclinical preheart failure (HF).** Overlapping histograms showing distribution of age at diagnosis of subclinical pre-HF (ie, impaired global longitudinal strain) in the HERZCHECK cohort (purple bars) and at first diagnosis of symptomatic HF in the entropy-balanced control cohort (red bars). Comparison of the weighted mean age at diagnosis (ie, 60.9±7.0 years for subclinical pre-HF and 67.5±6.4 years for symptomatic HF) demonstrates that the proposed screening yields ≈6.7 years of time gained for prevention of progression from subclinical pre-HF to symptomatic HF.

The weighted mean age at diagnosis of subclinical pre-HF in the HERZCHECK subgroup and at HF incidence in the entropy-balanced control cohort was 60.9±7.0 years and 67.5±6.4 years, respectively. Thus, subclinical pre-HF in the HERZCHECK subgroup was detected 6.7 years (95% CI, 6.11–7.25 years; *P*<0.001) earlier than symptomatic HF in the entropy-balanced control cohort (Figure 4). Results from subgroup and sensitivity analyses after entropy balancing are reported in Tables S7 and S8. Results before entropy balancing are reported in Tables S9 and S10.

## Discussion

Whereas the concept of subclinical pre-HF (stage B HF) as a precursor of symptomatic HF is well established in US HF guidelines, it remains largely unrecognized in their European counterparts.^13,14^ Data from 2 randomized controlled trials demonstrated that early identification of subclinical pre-HF can reduce the incidence of symptomatic HF, if followed by targeted preventive interventions, and indicate that adopting a similar approach in routine clinical care for the at-risk population could have a significant beneficial impact on the immense socioeconomic burden of HF.^3,4^

HERZCHECK herein presents the blueprint for a simple, standardized, GLS-based CMR screening approach for subclinical pre-HF that was developed based on previous evidence for the strong association of impaired GLS with incident symptomatic HF, as well as the high accuracy and reproducibility of CMR, even in individuals with typically challenging acoustic conditions posing problems in echocardiography.^5–8,15–17^ Systematic application of this screening approach in a representative cohort of 4509 participants with established cardiovascular risk factors from rural and underresourced regions in Germany confirmed its feasibility and demonstrated that around 23% (ie, almost one-fourth) of the at-risk population has traceable signs of subclinical pre-HF.

These findings are of great importance, as current estimates of the prevalence of subclinical pre-HF are highly variable, depending on specific participant selection and the diagnostic criteria used. Although most available studies defined subclinical pre-HF more broadly, using different selections of the diagnostic criteria for stage B HF outlined in the US HF guidelines, Russo and colleagues previously reported the prevalence of subclinical pre-HF assessed solely by impairments in GLS. Using a numerically comparable GLS cutoff (ie, −14.7%), they reported a prevalence of impaired GLS of 16.8% among those with preserved LVEF.^13,18^ Potential reasons for the slightly lower prevalence of subclinical pre-HF reported in the study by Russo and colleagues compared with HERZCHECK are manifold. For instance, Russo and colleagues used a more urban population from the Northern Manhattan Study, which could have affected the prevalence estimate, given that HF is generally known to be more common in structurally weak areas where access to specialists is low.^1,2,18^ A second reason that requires attention is that Russo and colleagues used echocardiography, whereas HERZCHECK utilized state-of-the-art CMR. While this and several other studies previously demonstrated that subclinical pre-HF can also be screened for using echocardiography, HERZCHECK deliberately used CMR due to its superior accuracy and reproducibility.^18,19^ Specifically, CMR offers the benefit of full myocardial coverage, allowing for strain analysis across all segments; higher spatial resolution with a better signal-to-noise ratio; and higher feasibility in obese patients with impaired acoustic conditions.^16–18,20^ In our trial, no participant was excluded due to morbid obesity. The last factor, in particular, could be a reason why patients with impaired GLS could have been missed using echocardiography but were detected when using CMR. This is also supported by the fact that Russo and colleagues had to exclude 125 (15%) of the 854 participants included in their study due to insufficient image quality for speckle-tracking analysis.^18^ In our trial, only 12 (0.3%) of 4666 participants had to be excluded due to reduced image quality.

In addition, by comparison with an entropy-balanced control cohort, we demonstrate that our screening approach allows for the detection of HF ≈7 years earlier than traditional methods. This earlier detection provides a crucial opportunity to initiate preventive measures aimed at delaying or even preventing the progression to symptomatic HF. However, a significant challenge remains: there is currently no clear consensus on the optimal management of these patients. Current guidelines emphasize risk factor optimization, yet such optimization does not necessitate advanced imaging for the detection of subclinical pre-HF.^13^ Thus, while early identification of subclinical pre-HF offers a potential clinical advantage, it remains uncertain whether it translates into tangible benefits for patients in terms of long-term outcomes. Notably, data from other trials evaluating diagnostic imaging as a primary intervention indicate that it could—even in the absence of targeted therapies. For instance, the SCOT-HEART trial evaluating coronary computed tomography angiography in patients with stable chest pain previously demonstrated that the knowledge of the presence of disease leads to better adherence to primary preventive measures and eventually improves clinical outcomes.^21^

Nevertheless, there is an urgent need for randomized controlled trials designed to evaluate which therapeutic interventions—be it lifestyle modifications (dietary adjustments, structured exercise programs), the use of wearable devices, or specific pharmacotherapy—can effectively delay or even prevent the progression to symptomatic HF. Specifically, it is crucial to determine whether all patients classified as having subclinical pre-HF should receive targeted therapy, or whether treatment should be reserved for specific subgroups—such as those with reduced GLS or other distinct criteria—to avoid overdiagnosis and overtreatment. Future research should also focus on refining risk stratification methods to better identify patients who are most likely to benefit from early intervention.

Other important aspects to consider when trying to assess the potential of the proposed screening algorithm to reduce the global burden of HF are its scalability in routine clinical care and its feasibility in different global settings. While both will ultimately need to be addressed in further studies, 2 specific features of the HERZCHECK screening are very promising in that regard. First, mobile CMR units can be deployed virtually anywhere in the world (high- and low-income countries), including the most remote areas, principally allowing for global adoption. Second, the GLS is a highly objective, quantifiable parameter that can be assessed (semi-) automatically. Therefore, expert interpretation is only required in cases of doubt and, as we demonstrated in this study, can then be provided via telemedicine. This will hopefully optimize the allocation and use of human resources by allowing highly trained experts to screen large numbers of individuals with excellent diagnostic accuracy. In addition, this approach might reduce the need for costly investments in smaller hospitals and help to set up networks with centralized expertise to provide broad access to high-end care at affordable costs for the healthcare system.

### Limitations

HERZCHECK was focused on a population from rural and underresourced regions of Germany. It is therefore unclear whether the results can be transferred to urban populations. The CMR protocol did not contain the administration of contrast agents, which would have been necessary for the full diagnostic workup of cardiomyopathies. However, it is due in particular to the absence of contrast agents, that the proposed CMR protocol can be performed under telemedical supervision without a physician on site. This enables widespread application of the proposed screening algorithm, even in the most remote areas lacking trained healthcare staff and hardware. Another limitation is the primarily cross-sectional design, which precludes direct assessment of long-term HF risk in the entire cohort. This was partly addressed by analyzing 1-year health insurance claims data in a subcohort. In addition, complementary insight into the diagnostic lead time compared with the standard of care was obtained using a matched and entropy-balanced control cohort from claims data. Matching and entropy balancing were performed without available information on the GLS and other clinical parameters, as these were not routinely reported in the claims data used for that purpose. However, due to the use of sophisticated statistical balancing methods including numerous covariates known to be associated with the cardiovascular risk, it can be assumed that the control cohort sufficiently matches the HERZCHECK subgroup considered for the respective analysis. Importantly, extended prospective follow-up studies allowing for definitive evaluation of the prognostic implications of CMR-based GLS are already planned. Furthermore, when using claims data, incident HF cases (CC) and adverse events may be misclassified. To account for such misclassification, different validation algorithms for incident HF were tested in sensitivity analyses presented in the Supplemental Material. In addition, the definition of adverse events was applied to both participants with and without subclinical pre-HF (ie, not affecting the differences). Moreover, the observed association of male sex with elevated risk for subclinical pre-HF should be interpreted with caution, as it may reflect residual or unmeasured confounding, and further analyses will be required to clarify the underlying mechanisms. Last, several risk factors included in the presented regression analyses were self-reported without an evaluation of more detailed information on their severity and treatment status, which may have influenced the ability to detect independent associations with subclinical pre-HF.

### Conclusions

**Figure 5.**
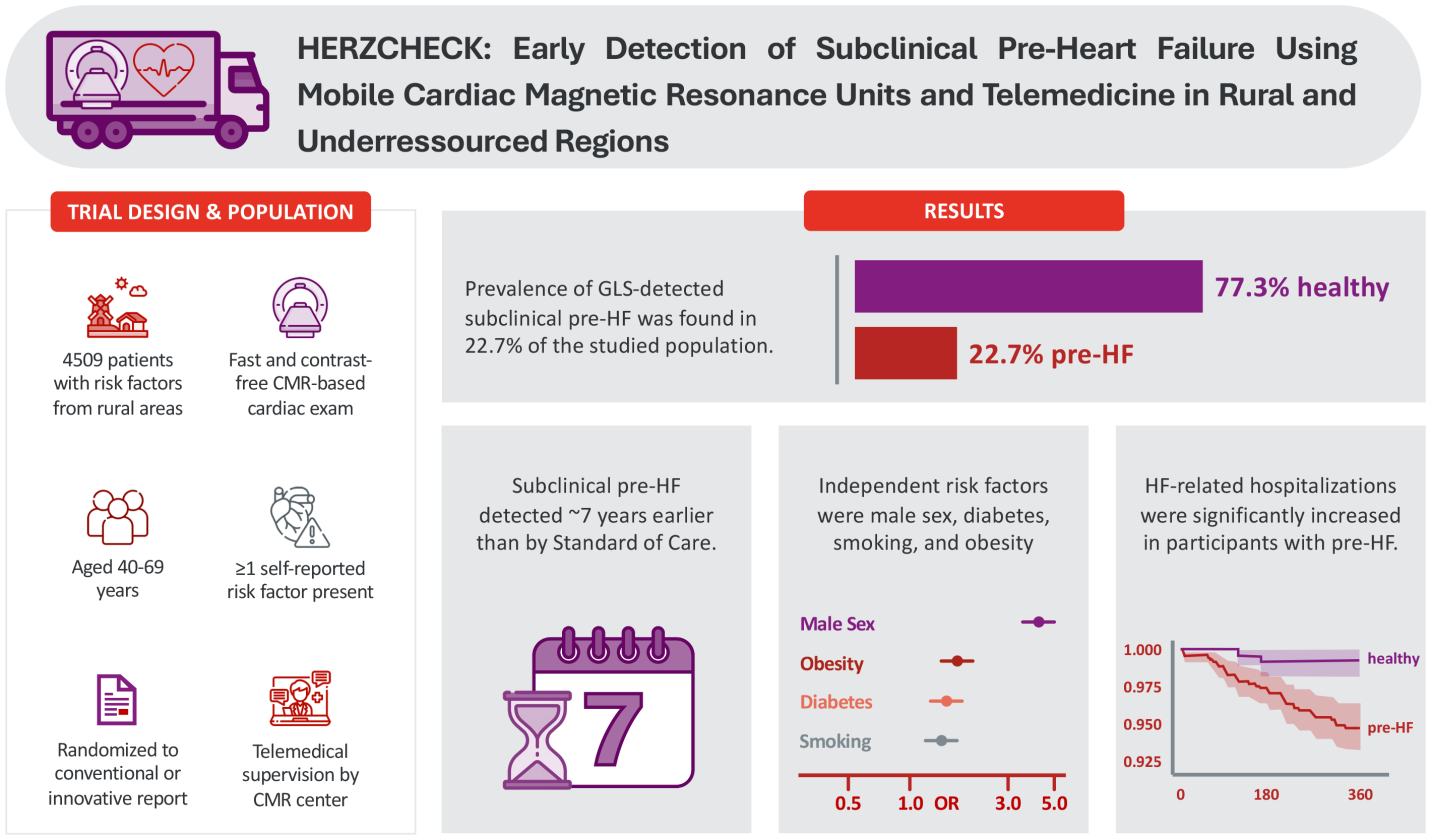
**Illustration visualizing characteristics of the study design and population, as well as key study results.** CMR indicates cardiac magnetic resonance; GLS, global longitudinal strain; HF, heart failure; and OR, odds ratio.

HERZCHECK provides a proposal for a contemporary, fully mobile, and telemedically supervised CMR-based screening approach for subclinical pre-HF that is tailored to the needs of the target population. This study shows that subclinical pre-HF affects approximately one fourth of the at-risk population in rural and underresourced regions and that the proposed GLS-based CMR screening identifies patients suitable for preventive interventions ≈7 years earlier than the current standard of care (Figure 5). In addition, HERZCHECK provides a sufficiently large database to distinguish cardiovascular risk factor constellations associated with particularly high risk for subclinical pre-HF. These constellations may help establish multiparametric definitions that will allow for even more specific selection of patients requiring improved patient care and could thus optimize the use of resources available to healthcare systems across the globe.

## ARTICLE INFORMATION

### Acknowledgments

The authors thank all study participants and referring physicians for their participation in this trial and acknowledge the hard work of the entire interdisciplinary team, without whose support HERZCHECK could not be conducted.

### Author Contributions

Drs Kelle, Thiede, Steen, Remppis, Huppertz, Simic, Reber, Landmesser, Frey, Pieske, Nagel, Schmidt, Falk, and Friede conceived and designed the study. Drs Kelle, Müller, Beyer, Thiede, Nolden, Tuit, Cvetkovic, Witt, and Reber collected the data. Drs Kelle, Müller, Beyer, Nolden, Wieditz, Tuit, Cvetkovic, Witt, Huppertz, Simic, Müller, Shukri, Stock, Kentenich, and Friede performed the analyses. Drs Kelle, Müller, Beyer, Wieditz, Kentenich, and Friede wrote the first draft of the manuscript. All authors critically reviewed the manuscript and provided intellectual input. All authors gave final approval for the publication of the manuscript.

### Disclosures

None.

### Supplemental Material

Supplemental Methods S1–S5

Tables S1–S10

Figures S1–S6

References 22–33

## Supplementary Material
