# Supplementary material for: HERZCHECK: Early Detection of Subclinical Heart Failure Using Mobile Cardiac Magnetic Resonance and Telemedicine in Rural and Underressourced Regions
Source: Circ Heart Fail. 2026 Aug 4;19(8):e013935. doi: 10.1161/CIRCHEARTFAILURE.125.013935 (PMC13480323; doi:10.1161/CIRCHEARTFAILURE.125.013935)
Supplement: Supplementary file 1 [file hhf-19-e013935-s001.pdf]

SUPPLEMENTAL MATERIAL

Table of Contents

1. SUPPLEMENTAL METHODS ... 2

Methods S1: Methodological details for matching and entropy-balancing of the control cohort ... 2
Methods S2: Details on the evaluation of adverse events in the HERZCHECK subgroup ... 4
Methods S3: Methodological details for evaluating time gained for prevention ... 5
Methods S4: Details on HF-diagnosis validation for evaluating time gained for prevention ... 6
Methods S5: Methodological details of sensitivity analyses and subgroup analyses for evaluating time gained for prevention ... 7

2. SUPPLEMENTAL FIGURES ... 8

Figure S1: Flowchart displaying the recruitment process (entire HERZCHECK cohort) ... 8
Figure S2: Regional distribution of subclinical pre-heart failure in the evaluated rural and underressourced areas of Germany ... 9
Figure S3: Venn-diagramm demonstrating the relationship between GLS-based subclinical pre-HF to preserved or reduced LVEF and normal or elevated NT-proBNP levels ... 10
Figure S4: Results of multivariable logistic regression analysis ... 11
Figure S5: Kaplan-Meier curves illustrating differences in HF-related event-free survival between participants with and without subclinical pre-HF ... 12
Figure S6: Comparison of the HERZCHECK subgroup and the control cohort before and after entropy balancing ... 13

3. SUPPLEMENTAL TABLES ... 14

Table S1: Definition of covariates for matching and balancing of the control cohort ... 14
Table S2: Cross-table demonstrating the relationship between GLS-based subclinical pre-HF and normal or elevated NT-proBNP levels ... 16
Table S3: Cross-table demonstrating the relationship between GLS-based subclinical pre-HF and preserved or reduced LVEF ... 17
Table S4: Results of adverse events in the HERZCHECK subgroup ... 18
Table S5: Comparison of baseline characteristics between the HERZCHECK subgroup and the matched control cohort before entropy balancing ... 19
Table S6: Comparison of baseline characteristics between the HERZCHECK subgroup and the matched control cohort after entropy balancing ... 20
Table S7: Results of sensitivity and subgroup analyses for the time gained for prevention after entropy balancing ... 21
Table S8: Results of sensitivity and subgroup analyses for the HF incidence after entropy balancing ... 22
Table S9: Results of sensitivity and subgroup analyses for the time gained for prevention before entropy balancing ... 23
Table S10: Results of sensitivity and subgroup analyses for the HF incidence before entropy balancing ... 24

1. Supplemental Methods

Methods S1: Methodological details for matching and entropy-balancing of the control cohort

The control cohort (CC) was obtained from claims data of the participating health insurance fund (AOK Nordost). It was built to have a comparable cardiovascular risk profile to a subgroup of the HERZCHECK cohort that included all participants diagnosed with subclinical pre-heart failure (pre-HF), who were insured at the AOK Nordost, lived in Mecklenburg-Western Pomerania (MVP) or Brandenburg (BB) at the end of the year of the cardiac magnetic resonance (CMR) (according to claims data), and consented to the analysis of their claims data. Therefore, first, the AOK Nordost conducted an exact matching (1:10) based on sex, age (in categories), region of residence (i.e., federal state and classification in urban, semi-urban, rural), and presence of at least one HF risk factor (i.e., hypertension, hypercholesterinemia, diabetes mellitus, chronic kidney disease, obesity) as defined in Supplemental Table S1. People were eligible for the CC if they were 40 to 70 years old, lived in MVP or BB at the end of 2013, and had a continuous insurance (i.e., at least one day per quarter) from January 1st 2012 to December 31st 2022 or death (whatever occurred first). Exclusion criterion was a HF diagnosis in 2012-2013 to ensure that only people at risk were considered. Second, the evaluators conducted a weighting procedure to further optimize covariate balance between the matched HERZCHECK participants and the CC. For the weighting procedure, entropy balancing, as described by Hainmueller et al.[22], was applied. Covariates included sex, age, region of residence, risk factors, psychological diseases, Charlson Comorbidity Index (CCI)[23,24], healthcare costs, participation in relevant disease management programs and statin intake as defined in Supplemental Table S1. All variables were determined based on claims data of the year 2013 for CC participants and of 12 months before the CMR for HERZCHECK participants. For HERZCHECK participants, the CMR date from the HERZCHECK trial data was linked to the claims data via the HERZCHECK trial pseudonym, birth year and sex.

A detailed definition of the criteria used for matching and entropy balancing is presented in Supplemental Table S1.

To identify participants with risk factors, the following ICD-10 codes were used[25]:

Hypertension:	I10.0 – I10.1, I10.9, I15.0 – I15.2, I15.8 – I15.9
Hypercholesterolaemia:	E78.0 – E78.1
Obesity:	E66.0 – E66.2, E66.8 – E66.9, E67.8, E68
Diabetes mellitus:	E10 – E14
Chronic kidney disease:	N18 – N19

To identify participants with psychological diseases the following ICD-10 codes were used[25]:

F00-F09:	Organic, including symptomatic, mental disorders
F10-F16, F18, F19:	Mental and behavioral disorders due to psychoactive substance use
F20-F29:	Schizophrenia, schizotypal and delusional disorders
F30-F33:	Mood (affective) disorders: manic episode, bipolar affective disorder, depressive episode, recurrent depressive disorder
F70-F79:	Mental retardation

Baseline characteristics (obtained from claims data) of the matched HERZCHECK and CC participants were reported using numbers and proportions for categorical variables, and means and standard deviations for continuous variables. Characteristics were compared between HERZCHECK participants and the CC using absolute standardized mean differences (ASMD). All claims data were thoroughly checked for integrity and plausibility. Duplicates were removed and negative costs (i.e., cancelled

inpatient cases that were not reimbursed by the AOK Nordost) were set to zero in consultation with the AOK Nordost.

Methods S2: Details on the evaluation of adverse events in the HERZCHECK subgroup

HF-Hospitalizations, HF and all-cause mortality within 12 months after the CMR were evaluated based on AOK Nordost claims data of all HERZCHECK participants who were followed after the CMR (i.e., 10% of participants without pre-HF, all participants with pre-HF) and were insured at the AOK Nordost. Thereby, HF hospitalizations were defined as hospital admission with a corresponding main or secondary diagnosis. HF was defined as any hospitalization with main or secondary diagnosis, or any new (i.e., no inpatient or outpatient diagnosis within 24 months before the CMR) secured outpatient diagnosis. A HF diagnosis was defined as ICD-10 Codes I11.0, I13.0, I13.2 and I50. To compare HERZCHECK participants with and without pre-HF, the CMR date and the screening result (GLS ≥ -15%: subclinical pre-HF or GLS < -15%: no subclinical pre-HF) from the HERZCHECK trial data were linked to the claims data via the HERZCHECK trial pseudonym, birth year and sex.

Events were presented as numbers and proportions for those with preserved GLS and those with subclinical pre-HF. In addition, survival was calculated and displayed using the Kaplan-Meier method[26,27]. Differences between participants with preserved GLS and those with subclinical pre-HF were assessed using the log-rank test. For all analyses, a p-value <0.05 indicated statistical significance. All analyses were conducted with R, version 4.4.1, R Foundation for Statistical Computing, Vienna, Austria. All claims data were thoroughly checked for integrity and plausibility. Duplicates were removed in consultation with the AOK Nordost.

Methods S3: Methodological details for evaluating time gained for prevention

To evaluate whether the HERZCHECK screening enables an early detection of subclinical pre-HF, it was tested for a difference in age at subclinical pre-HF/HF detection between the matched HERZCHECK and CC participants. Therefore, claims data of the AOK Nordost were collected for the CC and HERZCHECK participants with subclinical pre-HF, who were insured at the AOK Nordost. Claims data included patient demographics, data on outpatient and inpatient care, and data on medication. The data covered a patient-specific period of 24 months prior to 12 months after the CMR for HERZCHECK participants and ranged from January 1st, 2012, to December 31st, 2022, or death (whichever occurred first) for the CC. For HERZCHECK participants, the CMR date from the HERZCHECK trial data was linked to the claims data via the HERZCHECK trial pseudonym, birth year and sex. Age at subclinical pre-HF detection was defined as age of HERZCHECK participants with subclinical pre-HF at the CMR. Age at HF detection was defined as age of CC participants at the first validated HF-diagnosis (i.e., incident HF-diagnosis) documented in the claims data. A definition of a validated HF-diagnosis is provided in the Supplemental Methods S4.

It was tested for a difference between age at subclinical pre-HF detection in the HERZCHECK participants and the age at HF detection in the CC using weighted t-test, with p-values presented. Further, the mean difference and corresponding 95% confidence interval were reported. In addition, to evaluate how many HERZCHECK participants with screen-detected subclinical pre-HF would have been diagnosed in SoC, the number and proportion of incident HF cases in the CC and the HF incidence rate per 1,000 patient years was calculated. Therefore, CC participants were followed from January 1st, 2014, to December 31st, 2022, or death (defined as end of the last insurance period before December 31st, 2022; whichever occurred first). For all analyses, a p-value <0.05 indicated statistical significance. To account for multiple testing, the Benjamini-Hochberg procedure was used. All analyses were conducted with R, version 4.4.1, R Foundation for Statistical Computing, Vienna, Austria. All claims data were thoroughly checked for integrity and plausibility. Duplicates were removed in consultation with the AOK Nordost.

Methods S4: Details on HF-diagnosis validation for evaluating time gained for prevention

To identify participants with incident HF, the following ICD-10 diagnoses were used[25]:

I50	Heart failure
I13.0	Hypertensive heart and renal disease with (congestive) heart failure
I13.2	Hypertensive heart and renal disease with (congestive) heart failure and renal failure
I11.0	Hypertensive heart with (congestive) heart failure

Due to possible incorrect or biased coding or a lack of recording of diagnoses[28], an internal diagnosis validation was conducted. A HF diagnosis was considered as validated in the following cases[29–31]:

≥ 1 inpatient main or secondary diagnosis
≥ 2 secured outpatient diagnoses within two quarters in one year
≥ 2 secured outpatient diagnoses by ≥ 2 health care providers within one quarter
1 secured outpatient diagnosis + HF related medication within the same or the following quarter
1 secured outpatient diagnosis + HF-telemonitoring by the diagnosing health care provider within the same or the following three quarters

Thereby, HF related medication included Angiotensin-Converting Enzyme Inhibitors (ACEI), Angiotensin II receptor blocker (ARB), beta blockers (BB), Diuretics. These substance groups were selected because they are recommended for almost all HF participants[32]. A prescription of these substances was defined via the following Anatomical Therapeutic Chemical (ATC) classification codes:

ACE-inhibitors:	C09A, C09B
ARB:	C09C, C09D
Beta blockers:	C07
Diuretics:	C03

HF-telemonitoring was defined via the Codes of the Doctors' Fee Scale (Einheitlicher Bewertungsmaßstab – EBM) 03325, 03326 and 13578-13587[28,30,33].

Methods S5: Methodological details of sensitivity analyses and subgroup analyses for evaluating time gained for prevention

Subgroup analyses were conducted to evaluate whether results differed according to sex, region of residence, and age (for evaluating HF incidence only).

To test the robustness of the results, the following sensitivity analyses were conducted (i) to vi) for evaluating time gained for prevention; i) and ii) for evaluating the HF incidence in the CC):

- i) requiring secondary inpatient diagnoses to be validated by an outpatient diagnosis
- ii) defining two outpatient diagnoses as validated only if documented in two consecutive quarters
- iii) including cases beginning before or ending after the baseline-period (instead of only cases beginning before the baseline-period) for calculating costs
- iv) using rounded age of HERZCHECK participants for entropy balancing
- v) conducting entropy balancing only for CC participants with incident HF
- vi) using more conservative age at diagnosis (i.e., assuming the birthday at the beginning of the birth month for the HERZCHECK participants and at the end of the birth month for the CC participants).

2. Supplemental Figures

Figure S1: Flowchart displaying the recruitment process (entire HERZCHECK cohort)

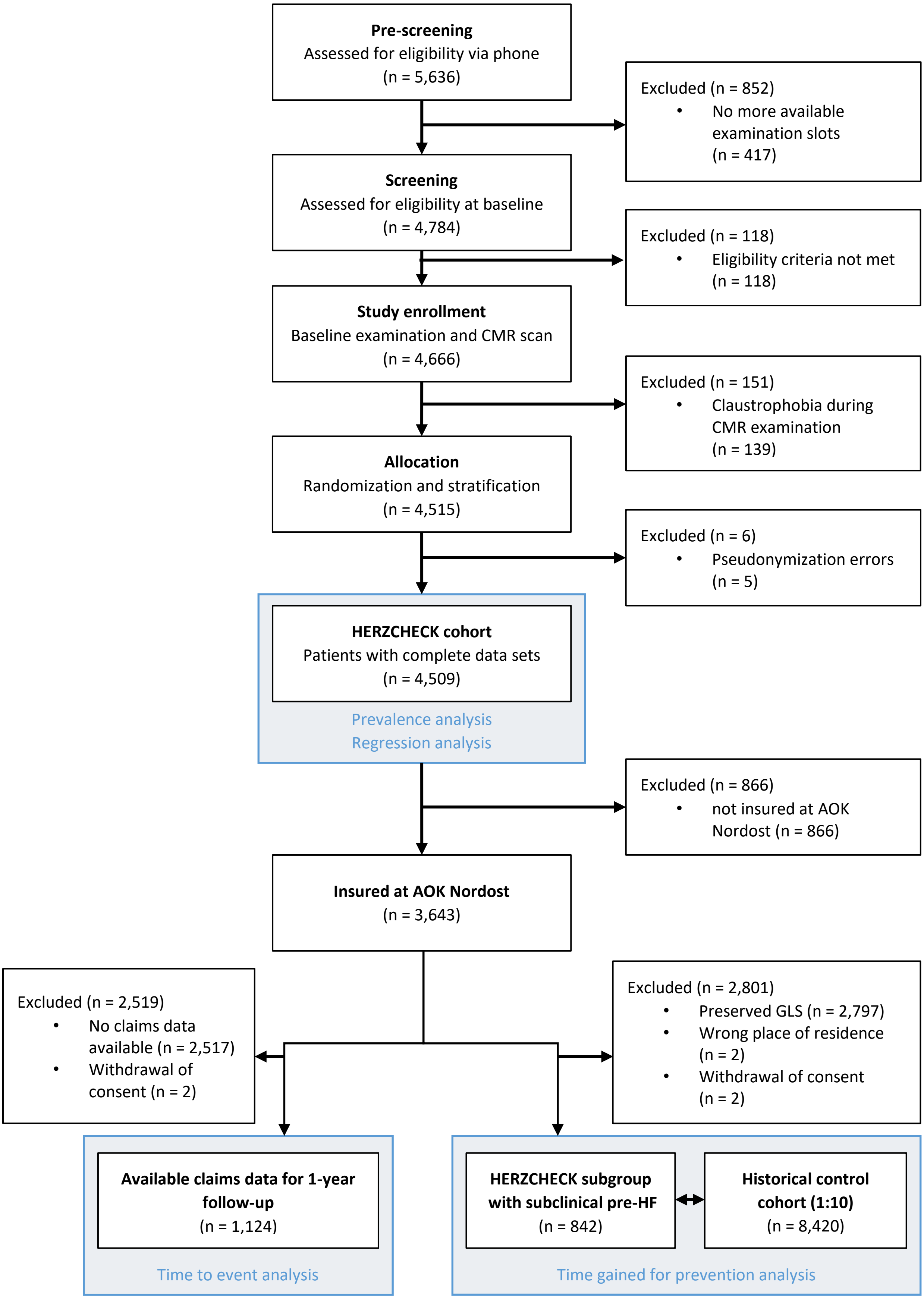

CMR = Cardiac Magnetic Resonance, GLS = Global Longitudinal Strain, HF = Heart Failure

Figure S2: Regional distribution of subclinical pre-heart failure in the evaluated rural and underressourced areas of Germany

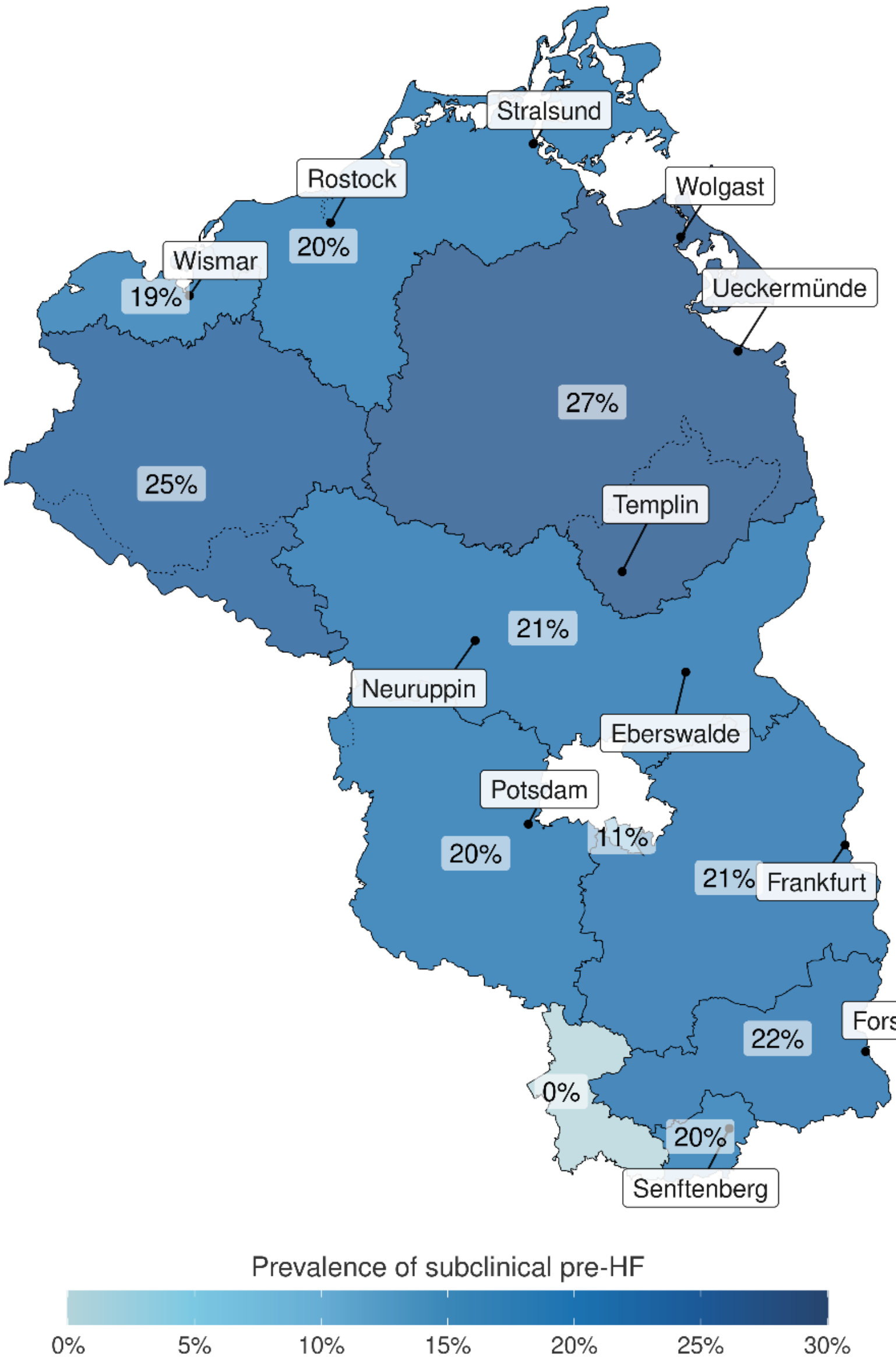

HF = Heart Failure, GLS = Global Longitudinal Strain

Figure S3: Venn-diagramm demonstrating the relationship between GLS-based subclinical pre-HF to preserved or reduced LVEF and normal or elevated NT-proBNP levels

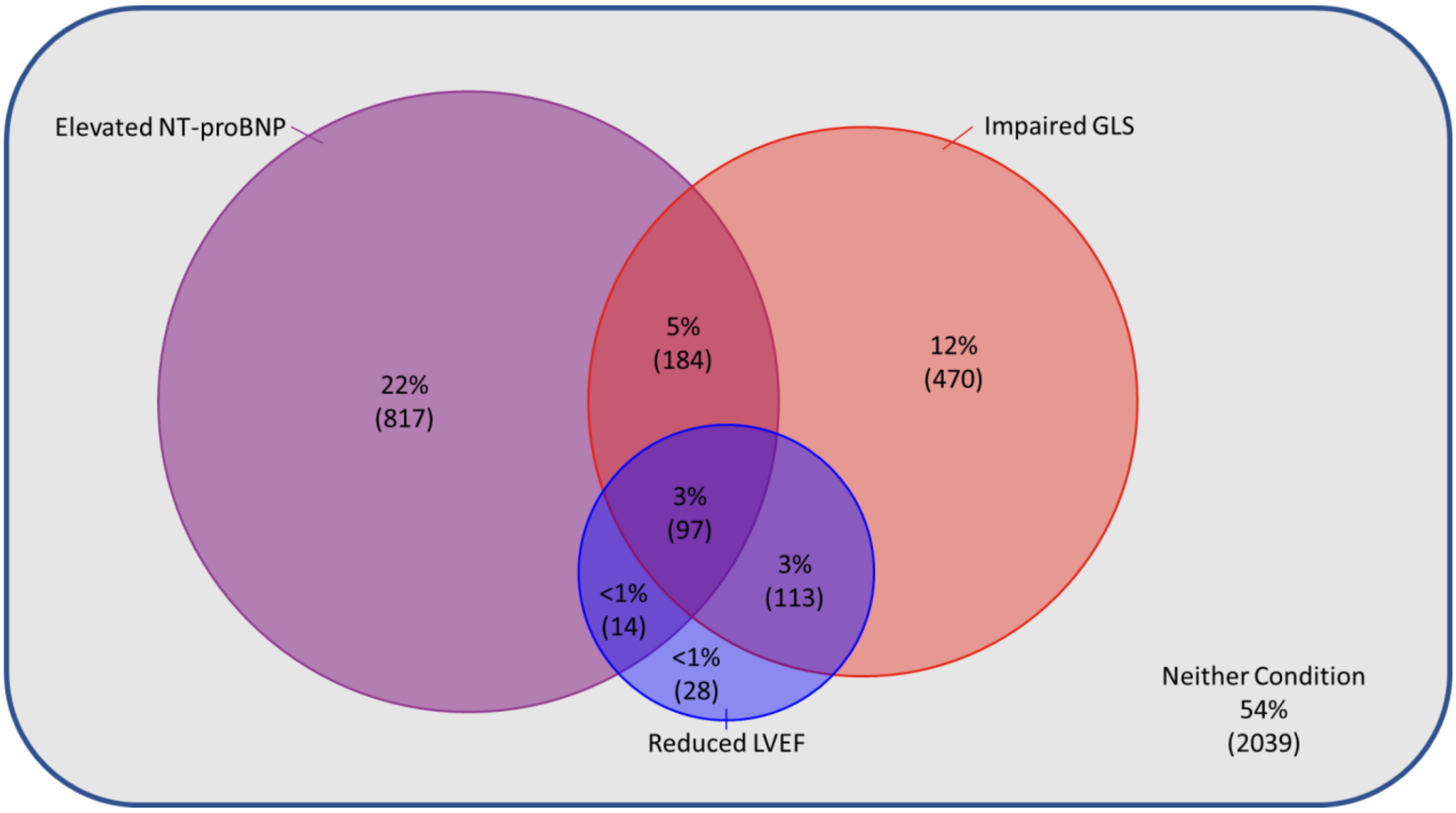

Overlap of abnormal NT-proBNP, impaired GLS, and reduced LVEF. Values represent absolute counts and percentages of the total cohort (N = 3,762). The sizes of the circles are proportional to group size; overlaps are schematic.

GLS = Global Longitudinal Strain, LVEF = Left Ventricular Ejection Fraction, NT-proBNP = N-terminal pro–B-type natriuretic peptide

Figure S4: Results of multivariable logistic regression analysis

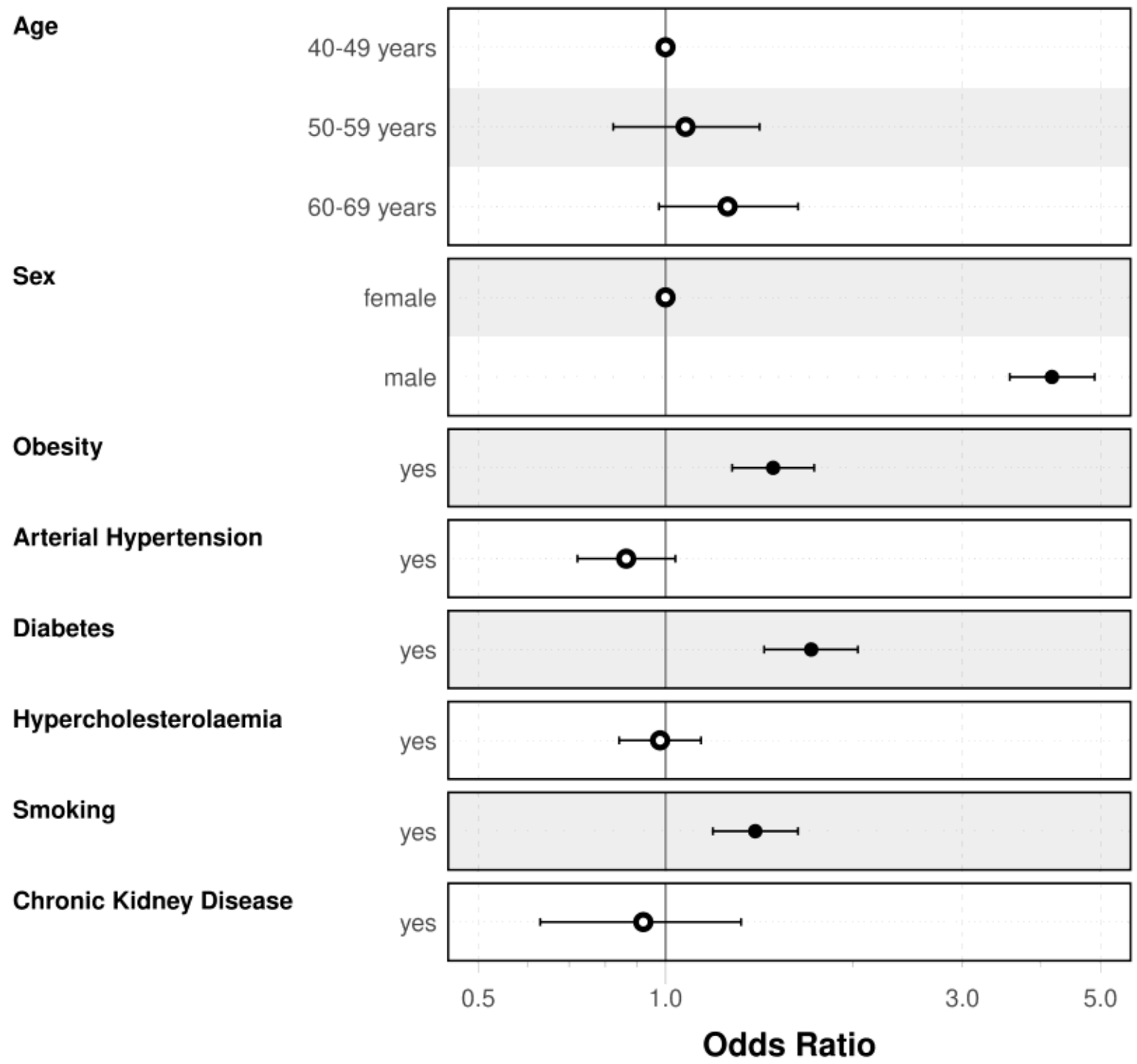

		OR	95% CI	p
Age	40-49 years	1.000		
	50-59 years	1.077	[0.824; 1.416]	0.591
	60-69 years	1.258	[0.976; 1.633]	0.080
Sex	female	1.000		
	male	4.178	[3.575; 4.893]	<0.001
Obesity	yes	1.489	[1.280; 1.733]	<0.001
Arterial Hypertension	yes	0.865	[0.722; 1.037]	0.116
Diabetes	yes	1.714	[1.441; 2.038]	<0.001
Hypercholesterolaemia	yes	0.980	[0.842; 1.140]	0.795
Smoking	yes	1.394	[1.192; 1.632]	<0.001
Chronic Kidney Disease	yes	0.921	[0.629; 1.323]	0.664

Results of the multivariable logistic regression analysis assessing the association of subclinical pre-heart failure with increasing age decades, sex, and individual cardiovascular risk factors. Results are given as OR with corresponding 95% CI.

CI = Confidence Interval, OR = Odds Ratio

Figure S5: Kaplan-Meier curves illustrating differences in HF-related event-free survival between participants with and without subclinical pre-HF

Please note that due to substantial interference of the intervention assessed in the integrated RCT, interpretability of differences is severely limited.

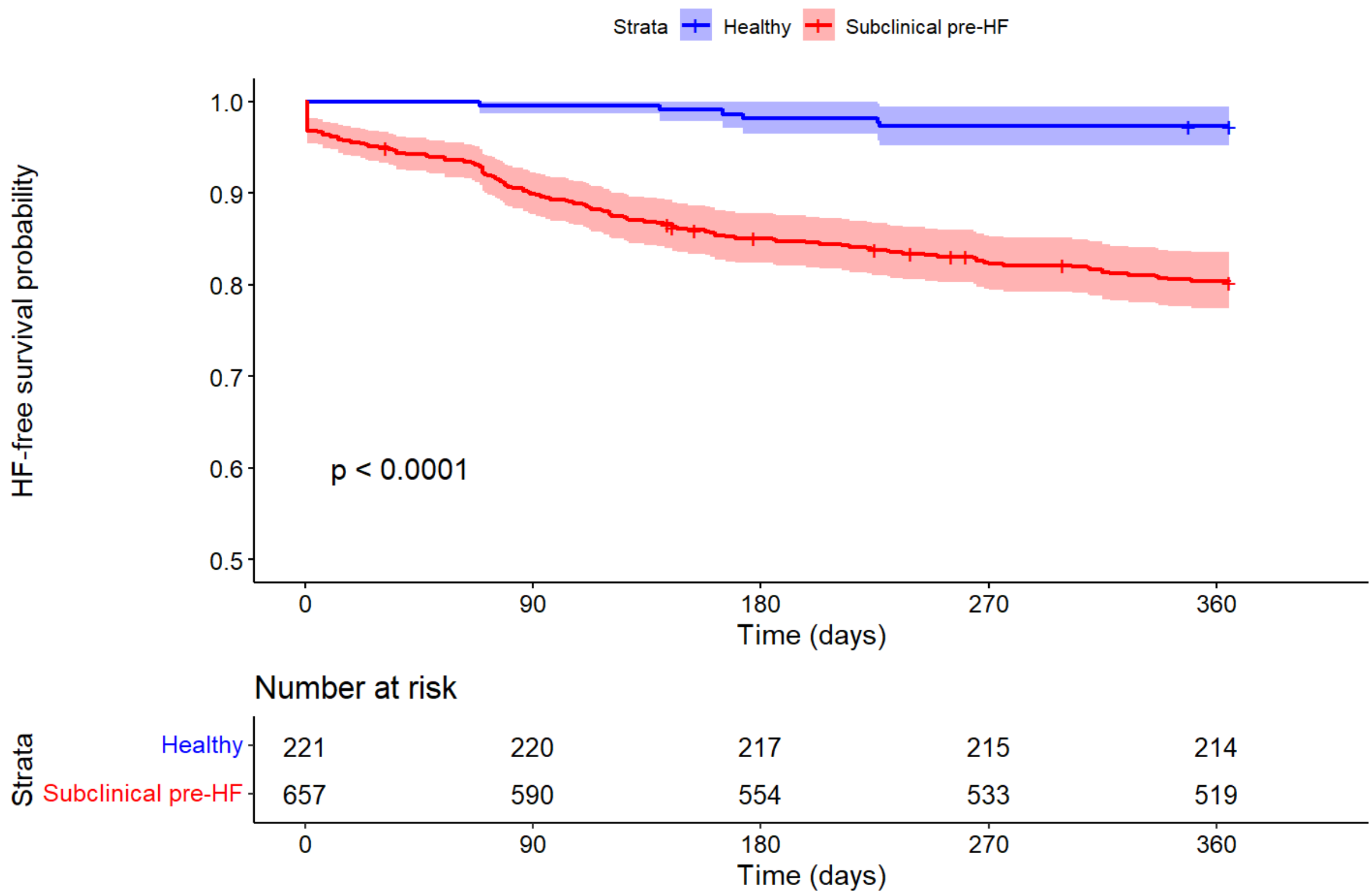

Kaplan-Meier curves showing a significant difference in the HF-free survival between HERZCHECK participants with and without subclinical pre-HF.
HF = Heart Failure

Figure S6: Comparison of the HERZCHECK subgroup and the control cohort before and after entropy balancing

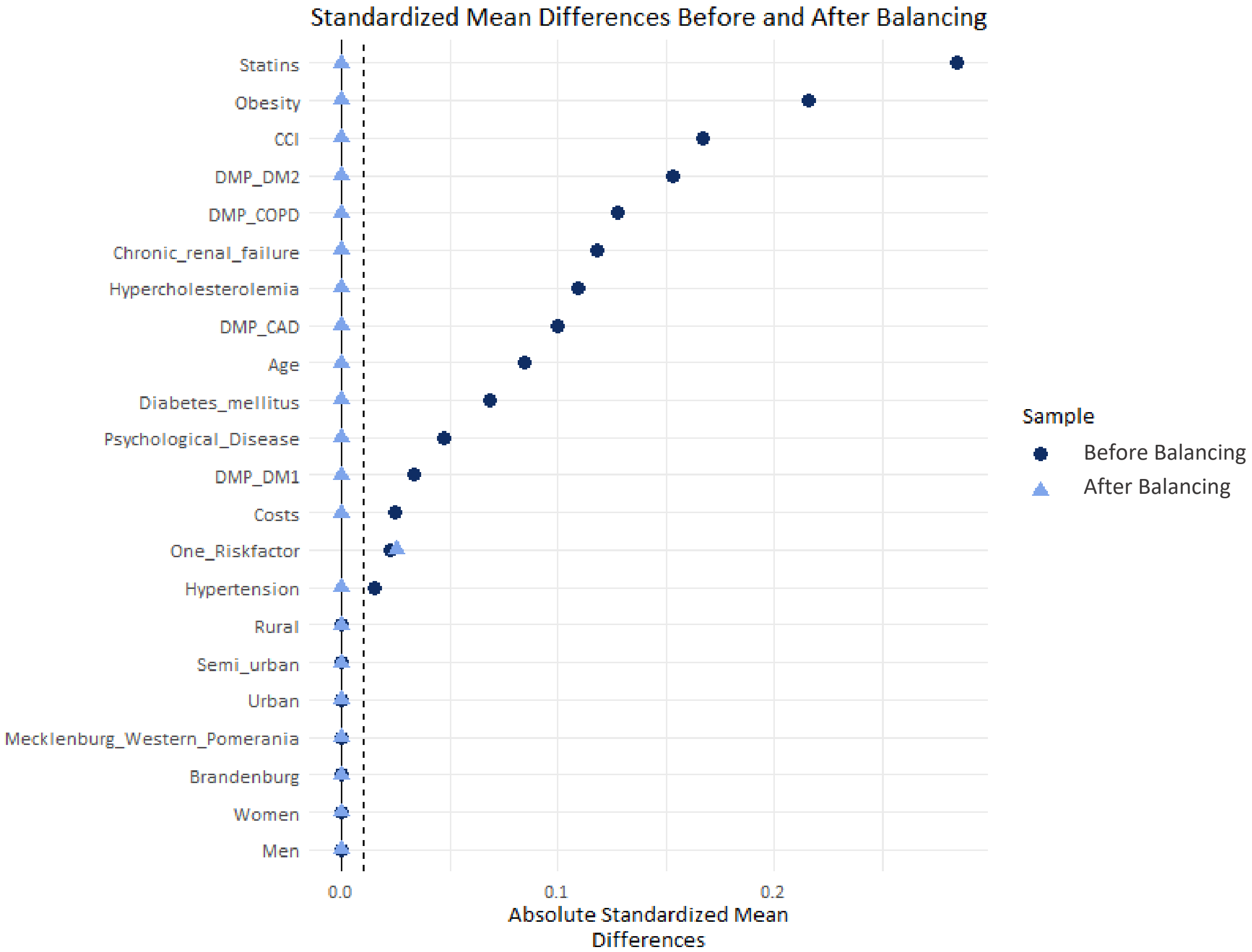

Covariate balance plot showing that, according to the absolute standardized mean differences, the HERZCHECK subgroup and the historical control cohort were comparable after entropy balancing.
CCI = Charlson Comorbidity Index, DMP = Disease Management Program, CAD = Coronary Artery Disease, DM1 = Diabetes Mellitus Type 1, DM2 = Diabetes Mellitus Type 2, COPD = Chronic Obstructive Pulmonary Disease

3. Supplemental Tables

Table S1: Definition of covariates for matching and balancing of the control cohort

Variable	Characteristics		Baseline period		Definition/Comment
	Matching	Balancing	HERZCHECK participants	control cohort	
Sex	Female/male	Female/male	Year of CMR	2013	Last sex documented in claims data
Age	Age classes<br>40-44<br>45-49<br>50-54<br>55-59<br>60-64<br>65-70	Continuous (40-70*)	Day of CMR	31.12.2013	Truncated age
Region of residence<br>Federal state<br>Classification	 BB/MVP<br>urban/semi-urban/rural	 BB/MVP<br>urban/semi-urban/rural	Year of CMR†	2013	Last region documented in claims data
Risk factors‡<br>Hypertension<br>Diabetes mellitus<br>Hypercholesterolemia<br>Obesity<br>Chronic kidney disease	At least one (yes/no)	Yes/no	12 months before CMR	2013	Inpatient main or secondary diagnosis or ≥ 2 secured outpatient diagnoses within two quarters in one year
Charlson comorbidity index‡ [23,24]	-	Continuous (1-29)	12 months before CMR	2013	Inpatient main or secondary diagnosis or ≥ 1 secured outpatient diagnosis

Variable	Characteristics		Baseline period		Definition/Comment
	Matching	Balancing	HERZCHECK participants	control cohort	
Psychological disease[‖]	-	Yes/no	12 months before CMR	2013	Inpatient main or secondary diagnosis or ≥ 1 secured outpatient diagnosis
Costs[‡]	-	Continuous	12 months before CMR	2013	Adjusted to 2023€
Statin intake	-	Yes/no	12 months before CMR	2013	Defined via ATC code C10AA
Participation in disease management program<br>Diabetes mellitus 1<br>Diabetes mellitus 2<br>Coronary artery disease<br>Chronic obstructive pulmonary disease	-	Yes/no	Year of CMR[†]	2013	At least one documented enrollment

[*] some participants were included in the study at age 69 years but turned 70 years before the first CMR; [†] no more detailed data was available; [‡] only diagnoses/costs of cases ending in the baseline period were considered; [‖] selection criterion for study invitation letters from AOK Nordost
ATC = Anatomical Therapeutic Chemical (classification), BB = Brandenburg, CMR = Cardiac Magnetic Resonance, MVP = Mecklenburg-Western Pomerania

Table S2: Cross-table demonstrating the relationship between GLS-based subclinical pre-HF and normal or elevated NT-proBNP levels

N = 3,762	**Elevated NT-proBNP (≥125 pg/mL)**	**Normal NT-proBNP (<125 pg/mL)**	**Total**
**Impaired GLS (≥ -15%)**	281 (7.5%)	583 (15.5%)	864 (23.0%)
**Preserved GLS (< -15%)**	831 (22.1%)	2,067 (54.9%)	2,898 (77.0%)
**Total**	1,112 (29.6%)	2,650 (70.4%)	3,762 (100%)

GLS = Global Longitudinal Strain, HF = Heart Failure, NT-proBNP = N-terminal pro B-type natriuretic peptide

Table S3: Cross-table demonstrating the relationship between GLS-based subclinical pre-HF and preserved or reduced LVEF

N = 4,509	**Reduced LVEF (<50%)**	**Preserved LVEF (≥50%)**	**Total**
**Impaired GLS (≥ -15%)**	244 (5.4%)	779 (17.3%)	1,023 (22.7%)
**Preserved GLS (< -15%)**	45 (1.0%)	3,441 (76.3%)	3,486 (77.3%)
**Total**	289 (6.4%)	4,220 (93.6%)	100%

GLS = Global Longitudinal Strain, HF = Heart Failure, LVEF = Left Ventricular Ejection Fraction

Table S4: Results of adverse events in the HERZCHECK subgroup

	**12 Months**		
	**Preserved GLS (n=282)**	**Subclinical pre-HF (n=842)**	**Total (n=1,124)**
Mortality; n (%)	1 (0.35%)	10 (1.19%)	11 (0.98%)
HF-Hospitalization; n (%)	2 (0.71%)	41 (4.87%)	43 (3.83%)
	**Preserved GLS (n=221)**	**Subclinical pre-HF (n=657)**	**Total (n=878)**
HF; n (%)	6 (2.71%)	129 (19.63%)	135 (15.38%)

GLS = Global Longitudinal Strain, HF = Heart Failure

Table S5: Comparison of baseline characteristics between the HERZCHECK subgroup and the matched control cohort before entropy balancing

Variable		Total (n=9,262)	HERZCHECK participants (n=842)	Control Cohort (n=8,420)	ASMD
Age; mean [SD]		60.9 [7.2]	60.4 [7.1]	61.0 [7.2]	0.0834
	≥60; n (%)	6,066 (65.5)	536 (63.7)	5,530 (65.6)	
Sex; n (%)					
	Male	6,578 (71.0)	598 (71.0)	5,980 (71.0)	<0.0001
	Female	2,684 (29.0)	244 (29.0)	2,440 (29.0)	<0.0001
Federal State; n (%)					
	Mecklenburg-Western Pomerania	4,125 (44.5)	375 (44.5)	3,750 (44.5)	<0.0001
	Brandenburg	5,137 (55.5)	467 (55.5)	4,670 (55.5)	<0.0001
Region; n (%)					
	Urban	1,716 (18.5)	156 (18.5)	1,560 (18.5)	<0.0001
	Semi-urban	3,960 (42.8)	360 (42.8)	3,600 (42.8)	<0.0001
	Rural	3,586 (38.7)	326 (38.7)	3,260 (38.7)	<0.0001
Risk Factors; n (%)					
	Hypertension	6,866 (74.1)	619 (73.5)	6,247 (74.2)	0.0154
	Hypercholesterolemia	1,510 (16.3)	171 (20.3)	1,339 (15.9)	0.1146
	Obesity	2,254 (24.3)	283 (33.6)	1,971 (23.4)	0.2274
	Diabetes mellitus	3,098 (33.4)	307 (36.5)	2,791 (33.1)	0.0696
	Renal insufficiency	560 (6.0)	77 (9.1)	483 (5.7)	0.1302
Number of risk factors; n (%)					
	≥1	7,831 (84.5)	718 (85.3)	7,113 (84.5)	0.0222
	1	3,506 (37.9)	262 (31.1)	3,244 (38.5)	
	2	2,610 (28.2)	245 (29.1)	2,365 (28.1)	
	3	1,323 (14.3)	149 (17.7)	1,174 (13.9)	
	4	367 (4.0)	52 (6.2)	315 (3.7)	
	5	25 (0.3)	10 (1.2)	15 (0.2)	
Psychological disease; n (%)		2,155 (23.3)	181 (21.5)	1,974 (23.4)	0.0467
Charlson comorbidity index; mean [SD]		1.7 [2.1]	2.1 [2.2]	1.7 [2.1]	0.1694
Statine intake; n (%)		2,399 (25.9)	324 (38.5)	2,075 (24.6)	0.3011
Disease Management Program; n (%)					
	Diabetes mellitus Type 1	42 (0.5)	6 (0.7)	36 (0.4)	0.0379
	Diabetes mellitus Type 2	2,047 (22.1)	239 (28.4)	1,808 (21.5)	0.1603
	Coronary artery disease	604 (6.5)	77 (9.1)	527 (6.3)	0.1084
	Chronic obstructive pulmonary disease	304 (3.3)	51 (6.1)	253 (3.0)	0.1472
Costs in €; mean [SD]		3,463.5 [8,794.6]	3,595.5 [5,917.9]	3,450.3 [9,032.2]	0.0190

ASMD = Absolute Standardized Mean Difference, CC = Control Cohort, SD = Standard Deviation

Table S6: Comparison of baseline characteristics between the HERZCHECK subgroup and the matched control cohort after entropy balancing

	Total (n=9,262)	HERZCHECK participants (n=842)	Control Cohort (n=8,420)	ASMD
Age; mean [SD]	60.4 [7.4]	60.4 [7.0]	60.4 [7.5]	<0.0001
≥ 60 years; n (%)	5,797 (62.6)	536 (63.7)	5,261 (62.5)	
Sex; n (%)				
Male	6,578 (71)	598 (71)	5,980 (71)	<0.0001
Female	2,684 (29)	244 (29)	2,440 (29)	<0.0001
Federal State; n (%)				
Mecklenburg-Western	4,125 (55.5)	375 (55.5)	3,750 (55.5)	<0.0001
Pomerania	5,137 (44.5)	467 (44.5)	4,670 (44.5)	<0.0001
Brandenburg				
Region; n (%)				
Rural	3,586 (38.7)	326 (38.7)	3,260 (38.7)	<0.0001
Semi-urban	3,960 (42.8)	360 (42.8)	3,600 (42.8)	<0.0001
Urban	1,716 (18.5)	156 (18.5)	1,560 (18.5)	<0.0001
Risk Factors; n (%)				
Hypertension	6,809 (73.5)	619 (73.5)	6,190 (73.5)	<0.0001
Hypercholesterolemia	1,881 (20.3)	171 (20.3)	1,710 (20.3)	<0.0001
Obesity	3,113 (33.6)	283 (33.6)	2,830 (33.6)	<0.0001
Diabetes mellitus	3,377 (36.5)	307 (36.5)	3,070 (36.5)	<0.0001
Renal insufficiency	847 (9.1)	77 (9.1)	770 (9.1)	<0.0001
Number of Risk Factors; n (%)				
≥1	7,975 (86.1)	718 (85.3)	7,257 (86.2)	0.0255
1	3,022 (32.6)	262 (31.1)	2,760 (32.8)	
2	2,580 (27.9)	245 (29.1)	2,335 (27.7)	
3	1,696 (18.3)	149 (17.7)	1,547 (18.4)	
4	629 (6.8)	52 (6.2)	577 (6.8)	
5	49 (0.5)	10 (1.2)	39 (0.5)	
Psychological disease; n (%)	1,991 (21.5)	181 (21.5)	1,810 (21.5)	<0.0001
Charlson comorbidity index; mean [SD]	2.1 [2.4]	2.1 [2.2]	2.1 [2.4]	<0.0001
Statine intake; n (%)	3,564 (38.5)	324 (38.5%)	3,240 (38.5)	<0.0001
Disease management program; n (%)				
Diabetes mellitus Type 1	66 (0.7)	6 (0.7)	60 (0.7)	<0.0001
Diabetes mellitus Type 2	2,629 (28.4)	239 (28.4)	2,390 (28.4)	<0.0001
Coronary artery disease	847 (9.1)	77 (9.1)	770 (9.1)	<0.0001
Chronic obstructive pulmonary disease	561 (6.1)	51 (6.1)	510 (6.1)	<0.0001
Costs in €; mean [SD]	3,595.5 [7,778.2]	3,595.5 [5,914.4]	3,595.5 [7,940.5]	<0.0001

ASMD = Absolute Standardized Mean Difference, SD = Standard Deviation

Table S7: Results of sensitivity and subgroup analyses for the time gained for prevention after entropy balancing

Scenario		Age; mean [SD]		Mean difference; years	95% CI	p-value*
		HERZCHECK participants	Control Cohort			
Base case						
**Base case**		60.9 [7.0]	67.5 [6.4]	***6.7***	6.11-7.25	< 0.001
Sensitivity analyses†						
**i) Diagnosis validation: validation of inpatient secondary diagnoses required**		60.9 [7.0]	67.8 [6.3]	**7.0**	6.30-7.55	<0.001
**ii) Diagnosis validation: outpatient diagnoses must be in consecutive quarters**		60.9 [7.0]	67.5 [6.4]	**6.6**	6.02-7.20	<0.001
**iii) Balancing: including also cases ending after baseline period**		60.9 [7.0]	67.5 [6.4]	**6.7**	6.04-7.25	<0.001
**iv) Balancing: Rounded Age**		60.9 [7.0]	67.9 [6.2]	**7.0**	6.42-7.56	<0.001
**v) Balancing: only incident cases**		60.9 [7.0]	65.1 [7.6]	**4.2**	3.54-4.96	<0.001
**vi) Age at diagnosis: conservative calculation**		60.9 [7.0]	67.5 [6.4]	**6.6**	6.01-7.13	<0.001
Subgroup analyses						
**Sex**	Male (n=6,578)	61.0 [7.2]	67.6 [6.4]	**6.6**	5.86 – 7.28	<0.001
	Female (n=2,684)	60.7 [6.7]	67.5 [6.3]	**6.8**	5.62 – 7.93	<0.001
**Region**	Urban (n=1,716)	60.0 [7.4]	68.2 [6.6]	**8.2**	6.85 – 9.64	<0.001
	Semi-urban (n=3,960)	60.9 [7.0]	67.4 [6.3]	**6.4**	5.50 – 7.35	<0.001
	Rural (n=3,586)	61.3 [6.9]	67.4 [6.4]	**6.1**	5.19 – 7.03	<0.001

* p-values were adjusted using Benjamini-Hochberg procedure, † refer to section methods S4 for methodological details,
CI = Confidence Interval, HF = Heart Failure, SD = Standard Deviation

Table S8: Results of sensitivity and subgroup analyses for the HF incidence after entropy balancing

Scenario		Observation time; years (mean per patient)	Incidence; n (%)	Incidence rate (per 1000 patient years)	Overall mortality; n (%)	Mortality in incident cases; n (%)
Base case						
**Base case**		60,457.9 (7.2)	2,177 (25.9)	36.0	1,665 (19.8)	613 (30.6)
Sensitivity analyses						
**i) Diagnosis validation: validation of inpatient secondary diagnoses required**		63,288.0 (7.5)	1,449 (17.2)	22.9	1,665 (19.8)	359 (27.0)
**ii) Diagnosis validation: outpatient diagnoses must be in consecutive quarters**		60,577.0 (7.2)	2,149 (25.5)	35.5	1,665 (19.8)	610 (30.9)
Subgroup analyses						
**Sex**	Male (n=5,980)	42,192.9 (7.1)	1,646 (27.5)	39.0	1,315 (22.0)	516 (33.7)
	Female (n=2,440)	18,261.1 (7.5)	546 (22.4)	29.9	331 (13.6)	97 (20.4)
**Region**	Urban (n=1,560)	11,603.3 (7.4)	328 (21.0)	28.3	336 (21.5)	117 (36.8)
	Semi-urban (n=3,600)	25,692.6 (7.1)	983 (27.3)	38.2	694 (19.3)	258 (29.0)
	Rural (n=3,260)	23,155.6 (7.1)	867 (26.6)	37.4	628 (19.3)	238 (29.8)
**Age**	Age 40-49 (n=710)	6,015.5 (8.5)	49 (6.9)	8.1	42 (5.9)	13 (25.5)
	Age 50-59 (n=2,180)	16,900.5 (7.8)	376 (17.2)	22.2	299 (13.7)	82 (22.6)
	Age 60-70 (n=5,530)	37,601.5 (6.8)	1,734 (31.4)	46.1	1,328 (24.0)	518 (32.6)

HF = Heart Failure

Table S9: Results of sensitivity and subgroup analyses for the time gained for prevention before entropy balancing

Scenario		Age; mean [SD]		Mean difference; years	95% CI	p-value[*]
		HERZCHECK participants	Control Cohort			
Base case						
**Base case**		60.9 [7.0]	68.1 [6.1]	7.2	6.69-7.77	< 0.001
Sensitivity analyses[†]						
**i) Diagnosis validation: validation of inpatient secondary diagnoses required**		60.9 [7.0]	68.4 [6.1]	7.5	6.92-8.08	< 0.001
**ii) Diagnosis validation: outpatient diagnoses must be in consecutive quarters**		60.9 [7.0]	68.1 [6.1]	7.2	6.7-7.76	< 0.001
**iii) Balancing: including also cases ending after baseline period**		60.9 [7.0]	68.1 [6.1]	7.2	6.69-7.77	< 0.001
**iv) Balancing: Rounded Age**		60.9 [7.0]	68.1 [6.1]	7.2	6.69-7.77	< 0.001
**v) Balancing: only incident cases**		60.9 [7.0]	68.1 [6.1]	7.2	6.69-7.77	< 0.001
**vi) Age at diagnosis: conservative calculation**		60.9 [7.0]	68.1 [6.1]	7.2	6.63-7.71	< 0.001
Subgroup analyses						
**Sex**	Male (n=6,578)	61.0 [7.2]	68.1 [6.2]	7.1	6.51-7.81	< 0.001
	Female (n=2,684)	60.7 [6.7]	68.2 [6.0]	7.5	6.48-8.59	< 0.001
**Region**	Urban (n=1,716)	60.0 [7.4]	68.4 [6.5]	8.4	6.97-9.77	< 0.001
	Semi-urban (n=3,960)	60.9 [7.0]	68.2 [6.0]	7.2	6.41-8.02	< 0.001
	Rural (n=3,586)	61.3 [6.9]	67.9 [6.2]	6.7	5.84-7.58	< 0.001

[*] p-values were adjusted using Benjamini-Hochberg procedure, [†] refer to section Methods S4 for methodological details,
CI = Confidence Interval, HF = Heart Failure, SD = Standard Deviation

Table S10: Results of sensitivity and subgroup analyses for the HF incidence before entropy balancing

Scenario		Observation time; years (mean per patient)	Incidence; n (%)	Incidence rate (per 1000 patient years)	Overall mortality; n (%)	Mortality in incident cases; n (%)
Base case						
**Base case**		61,344.4 (7.3)	2,005 (23.8)	32.7	1,654 (19.6)	613 (30.6)
Sensitivity analyses						
**i) Diagnosis validation: validation of inpatient secondary diagnoses required**		63,866.8 (7.6)	1,329 (15.8)	20.8	1,654 (19.6)	359 (27.0)
**ii) Diagnosis validation: outpatient diagnoses must be in consecutive quarters**		61,460.3 (7.3)	1,976 (23.5)	32.2	1,654 (19.6)	610 (30.9)
Subgroup analyses						
**Sex**	Male (n=5,980)	42,452.9 (7.1)	1,530 (25.6)	36.0	1,383 (23.1)	516 (33.7)
	Female (n=2,440)	18,891.5 (7.7)	1,976 (19.5)	25.1	271 (11.1)	97 (20.4)
**Region**	Urban (n=1,560)	11,540.1 (7.4)	318 (20.4)	27.6	349 (22.4)	117 (36.8)
	Semi-urban (n=3,600)	26,322.3 (7.3)	889 (24.7)	33.8	659 (18.3)	258 (29.0)
	Rural (n=3,260)	23,482.0 (7.2)	798 (24.5)	34.0	646 (19.8)	238 (29.8)
**Age**	Age 40-49 (n=710)	5,980.3 (8.4)	51 (7.2)	8.5	53 (7.5)	13 (25.5)
	Age 50-59 (n=2,180)	17,021.2 (7.8)	363 (16.7)	21.3	296 (13.6)	82 (22.6)
	Age 60-70 (n=5,530)	38,342.9 (6.9)	1,591 (28.8)	41.5	1,305 (23.6)	518 (32.6)

HF = Heart Failure
